# The Q-System as a Synthetic Transcriptional Regulator in Plants

**DOI:** 10.3389/fpls.2020.00245

**Published:** 2020-03-11

**Authors:** Ramona Persad, D. Nikki Reuter, Lezlee T. Dice, Mary-Anne Nguyen, Stephen B. Rigoulot, Jessica S. Layton, Manuel J. Schmid, Magen R. Poindexter, Alessandro Occhialini, C. Neal Stewart, Scott C. Lenaghan

**Affiliations:** ^1^Department of Food Science, The University of Tennessee, Knoxville, Knoxville, TN, United States; ^2^Center for Agricultural Synthetic Biology, The University of Tennessee, Knoxville, Knoxville, TN, United States; ^3^Department of Plant Sciences, The University of Tennessee, Knoxville, Knoxville, TN, United States

**Keywords:** Q-system, plant synthetic biology, gene expression, genetic circuits, metabolic engineering

## Abstract

A primary focus of the rapidly growing field of plant synthetic biology is to develop technologies to precisely regulate gene expression and engineer complex genetic circuits into plant chassis. At present, there are few orthogonal tools available for effectively controlling gene expression in plants, with most researchers instead using a limited set of viral elements or truncated native promoters. A powerful repressible-and engineerable-binary system that has been repurposed in a variety of eukaryotic systems is the Q-system from *Neurospora crassa*. Here, we demonstrate the functionality of the Q-system in plants through transient expression in soybean (*Glycine max*) protoplasts and agroinfiltration in *Nicotiana benthamiana* leaves. Further, using functional variants of the QF transcriptional activator, it was possible to modulate the expression of reporter genes and to fully suppress the system through expression of the QS repressor. As a potential application for plant-based biosensors (phytosensors), we demonstrated the ability of the Q-system to amplify the signal from a weak promoter, enabling remote detection of a fluorescent reporter that was previously undetectable. In addition, we demonstrated that it was possible to coordinate the expression of multiple genes through the expression of a single QF activator. Based on the results from this study, the Q-system represents a powerful orthogonal tool for precise control of gene expression in plants, with envisioned applications in metabolic engineering, phytosensors, and biotic and abiotic stress tolerance.

## Introduction

Plant synthetic biology is focused on the development of sophisticated molecular tools that enable precision metabolic engineering by allowing coordinated, tunable, inducible, and spatiotemporally regulated gene expression. While numerous tools and strategies exist for controlling gene expression in mammalian, bacterial, and yeast systems, plant synthetic biology currently suffers from a relative dearth of such tools. Even for simple chemically inducible expression, there are few robust systems in plants, with ethanol (Felenbok, 1991; Felenbok et al., 2001), dexamethasone (Gatz et al., 1992; Samalova et al., 2005, 2019), β*-*estradiol (Zuo et al., 2000), ecdysone (Martinez et al., 1999), tetracycline (Gatz and Quail, 1988; Gatz et al., 1992; Weinmann et al., 1994), and 3-methylcholanthrene (Kodama et al., 2007) leading the charge. With regards to controlling the stoichiometry or coordinating the expression of multiple genes, there are simply no ubiquitous, well validated tools for achieving these goals in plants. As such, it is necessary to look to other organisms to identify orthogonal tools that can be repurposed for use in plants. One such system, the quinic acid (*qa*) gene cluster of the fungus *Neurospora crassa*, referred to as the Q-system, has shown significant promise as an orthogonal tool for controlling gene expression in a variety of organisms, including *Drosophila* (Potter et al., 2010), *Caenorhabditis elegans* (Wei et al., 2012), zebrafish (Subedi et al., 2014), mammals (Potter et al., 2010; Fitzgerald et al., 2017), and more recently the plant *Nicotiana benthamiana* (Reis et al., 2018). Demonstration of the Q-system in plants represents a key first step, but further validation is necessary to harness the true potential of the system as a tool for plant synthetic biology.

**FIGURE 1:**
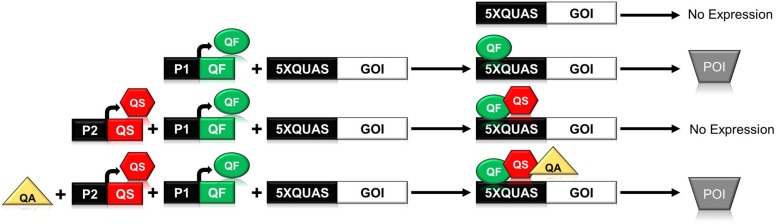
Schematic illustrating the function of the components of the *Neurospora crassa* Q-system. The Q-system is a multi-component system composed of a transcriptional activator (QF), repressor (QS), and a small molecule inducer, quinic acid (QA), that removes the effect of the repressor. As shown above, binding of QF to the QUAS promoter sequence results in expression of the gene of interest (GOI) leading to production of the protein of interest (POI). If QS is expressed in concert with QF, then QF is repressed and no gene expression is observed. If all three components, QF, QS, and QA are present, then the system is once again activated.

In *N. crassa*, the *qa* gene cluster consists of five structural and two regulatory genes that are involved in the catabolism of quinic acid for use as a carbon source (Giles et al., 1985; Tang et al., 2011). The synthetic Q-system utilizes three main components from the *qa* gene cluster: the QUAS transcription factor binding site, and the two regulatory genes *qa-1F* (quinate-1F) and *qa-1S* (quinate-1S), which encode the transcriptional activator QF, and its repressor QS (Huiet and Giles, 1986; Baum et al., 1987). The complete Q-system constitutes a repressible binary system, wherein the binding of QF to a minimal promoter containing the QUAS sequence triggers gene expression (Figure 1). Secondary binding of QS to QF inhibits the activity of QF; however, the addition of quinic acid to the system removes the inhibitory effect of QS (Potter et al., 2010; Riabinina et al., 2015). In early iterations of the system it was noticed that high levels of QF were toxic to *Drosophila* (Potter et al., 2010; Riabinina et al., 2015). To ameliorate toxicity, two QF variants (QF2 and QF2w) were designed and tested to eliminate the toxic effect while maintaining control over gene expression (Riabinina et al., 2015). Both QF2 and QF2w variants differ from the original QF by the deletion of their middle domain (MD), with the QF2w variant mutated in its last two C-terminal amino acids to produce a positive charge that reduces activity (Riabinina et al., 2015, 2019; Riabinina and Potter, 2016).

In this study, we validated the functionality of the Q-system in plants through *in vitro* assays in soybean and *in planta* assays in *N. benthamiana*. In particular, we investigated the impact of the promoter driving QF on subsequent reporter gene expression, coordination of multiple genes under a single QF activator, and tunability of gene expression level through variation in QUAS copy number. Practically, we also demonstrated the ability of the Q-system to amplify a reporter signal from a weak promoter, which has significant implications for standoff detection of phytosensors.

## Materials and Methods

### Expression Vector Construction

**FIGURE 2:**
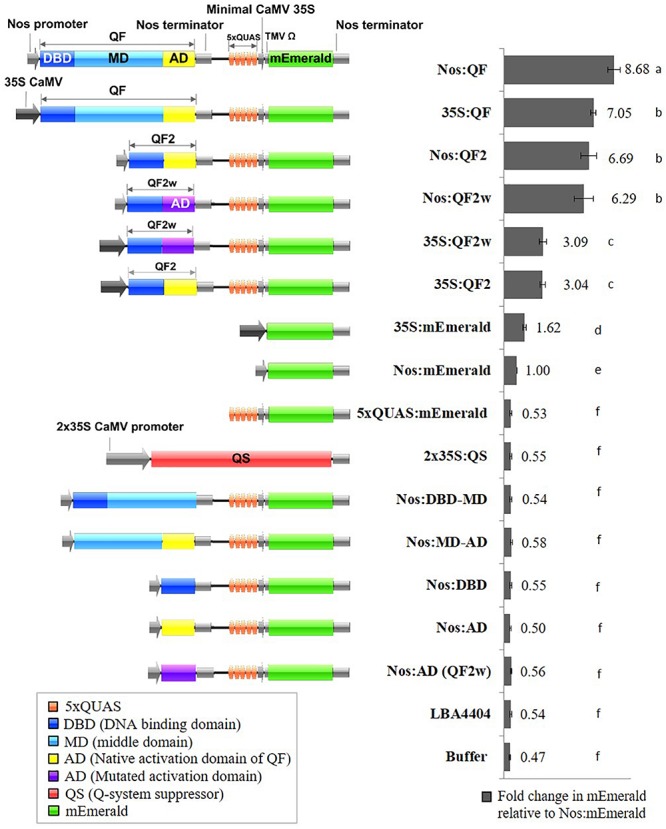
Schematic representation of Q-system constructs and the fold change in mEmerald fluorescence relative to Nos:mEmerald. Spectrofluorescence readings taken at 509 nm, 72 h post *Agrobacterium* infiltration of *N. benthamiana* leaves. Statistical significant differences determined using one-way ANOVA with *post hoc* analysis using Tukey HSD: groups with different letters show a significant difference (*p* < 0.05). Data represent mean ± standard error of three independent experiments (*n* = 3). Three technical replicates were collected for each biological replicate to account for positional error.

Genetic sequences for the *qa-1F* gene (QF) (NCBI Gene ID: 3875756), its variants (QF2 and QF2w), the *qa-1S* gene (QS) (NCBI Gene ID: 3875776), and 5xQUAS were obtained from Potter et al. (2010). Vector construction was carried out using the binary plant expression vector pMTV as the backbone. The following DNA fragments were synthesized by GeneArt and used to assemble the final vectors: *Pac*I-5xQUAS:mEmerald-*Asc*I, *Mfe*I-Nos:QF-*Spe*I, *Mfe*I-Nos:QF2-*Spe*I, *Mfe*I-Nos:QF2w-*Spe*I, *Mfe*I-35S:QF-*Spe*I, *Mfe*I-35S:QF2-*Spe*I, *Mfe*I-35S:QF2w-*Spe*I, and *Eco*RI-QS-*Asc*I (Supplementary Table S1). Sequential cloning was conducted to first introduce the *Pac*I-5xQUAS:mEmerald-*Asc*I cassette into the pMTV vector to make pMTV-5xQUAS:mEmerald. Next the *Mfe*I-X-*Spe*I constructs were cloned into pMTV-5xQUAS:mEmerald to generate the constructs pMTV-Nos:QF, pMTV-Nos:QF2, pMTV-Nos:QF2w, pMTV-35S:QF, pMTV-35S:QF2, pMTV-35S:QF2w. An additional construct was generated to assess suppression of QF by QS by cloning *Spe*I-2 × 35S-QS-*Pac*I into pMTV:Nos:QF. In addition, several control constructs with different combinations of QF transcription factor domains [DNA binding domain (DBD), MD, and activation domain (AD)] were produced using PCR and conventional restriction digest cloning and named: Nos:DBD-MD, Nos:MD-AD, Nos:DBD, Nos:AD, and Nos:AD (QF2w). The QS suppressor vector was assembled by cloning the *Eco*RI-QS-*Asc*I fragment into the pMTV-2 × 35S-2 × 35S binary vector resulting in expression of the QS suppressor being driven by the 2 × 35S promoter. To determine baseline expression of the reporter genes from the same promoters driving the QF transcription factor, Nos:mEmerald and 35S:mEmerald constructs were assembled. It should be noted that a weak 5’UTR was used for all promoters driving QF expression, and the Nos and 35S:mEmerald controls, to prevent excessive levels of reporter gene expression that could lead to saturation of the analytical techniques. The 5xQUAS:mTagBFP2 and 5xQUAS:mScarlet-I constructs were produced by PCR, restriction digestion, and cloning of fragments *Nco*I-mScarlet-I-*Asc*I and *Nco*I-mTagBFP2-*Asc*I into the pMTV-5xQUAS:mEmerald vector. Finally, multiple QUAS repeats (10xQUAS, 15xQUAS, 20xQUAS, and 25xQUAS) were synthesized by GeneArt and cloned by restriction digestion into pMTV:Nos:QF to produce the constructs: pMTV-Nos:QF:10xQUAS:mEmerald, pMTV-Nos:QF:15xQUAS: mEmerald, pMTV-Nos:QF:20xQUAS:mEmerald and pMTV-Nos:QF:25xQUAS:mEmerald. All constructs were confirmed by sequencing and schematic block diagrams of constructs were generated using the program Illustrator for Biological Sequences (IBS) (Liu et al., 2015; Figure 2).

### Plant Material

*Nicotiana benthamiana* plants were germinated and transferred to four-inch square pots after cotyledons were fully expanded. Transient expression experiments were performed using 4-week-old plants (post transplantation), grown in a 23–25°C growth chamber at 300 μE m^–2^ s^–1^, under a 16-h light/8-h dark cycle.

### *Agrobacterium* Transient Expression Assays

*Agrobacterium tumefaciens* strain LBA4404 was grown overnight in YEP media (5 g/L NaCl, 10 g/L peptone, and 10 g/L yeast extract) with 50 μg/ml rifampicin antibiotic for selection. Competent cells were prepared by washing cells in ice cold 10 mM calcium chloride and transformed with a single expression construct by incubating 1 μg of DNA with cells for 1 min in liquid nitrogen followed by 5 min at 37°C. Cells were allowed to recover for 3 h at 28°C on a 225 rpm shaking platform and transferred to selection plates. Cultures were grown overnight from a single colony, and 100 μM of acetosyringone was added to cultures 1 h before removing from the shaking platform. Cells were pelleted by centrifuging at 3000 × *g* for 15 min and resuspended in *Agrobacterium* infiltration buffer (10 mM MgCl_2_, 10 mM MES, 100 μM acetosyringone, pH 5.6). *Agrobacterium* infiltration solution was incubated at room temperature for 3 h prior to leaf infiltration. Four-week-old *N. benthamiana* plants were used for infiltration with *Agrobacterium* resuspended at an optical density (O.D. 600) of 0.5. *Agrobacterium* was delivered into leaves either using syringe infiltration (Norkunas et al., 2018), or by vacuum infiltration. For vacuum infiltration, plants were fully submerged into magenta boxes (Phytotech) containing *Agrobacterium* infiltration solution and placed into a 20 L aluminum vacuum chamber (Best Value Vacs). While plants were submerged, a vacuum pressure of approximately -84 kPa was applied three consecutive times. After infiltration, plants were dried using filter paper and returned to normal growth conditions. Three independent agroinfiltration experiments were performed, each with a single biological replicate per construct (*n* = 3). For each of the three biological replicates, three spectrofluorescence readings were taken on the second fully expanded leaf from the apical meristem.

### Fluorescence Spectroscopy

Fluorescence spectroscopy was performed 72 h after *Agrobacterium* infiltration. Fluorescence excitation and emission measurements were carried out using a Fluorolog^®^-3 spectrofluorometer according to the manufacturer’s instructions (HORIBA Scientific, version 3.8.0.60). For mEmerald, an excitation of 475 nm and emission range of 495–595 nm was used to obtain fluorescence emission peaks.

### RNA Extraction, cDNA Synthesis, and Quantitative Reverse Transcriptase-PCR

In order to analyze the suppression of QF by QS, tissue samples of leaves agroinfiltrated with Nos:QF:5xQUAS:mEmerald and Nos:QF:2 × 35S:QS:5xQUAS:mEmerald were collected for RNA isolation and analysis of both the QF transcription factor and the reporter gene. qRT-PCR was performed using tissue samples collected from the aforementioned three independent agroinfiltration experiments. Each independent experiment of qRT-PCR (*n* = 3) includes one biological replicate performed with three technical replicates. Total RNA was isolated using plant RNA purification reagent (Invitrogen). A volume of 500 μl of plant RNA purification reagent was added to approximately 12 mg of ground plant tissue, vortexed, and incubated at room temperature for 5 min. Thereafter, 100 μl of 5 M NaCl was added to each sample and mixed, then 300 μl of chloroform-isoamylalcohol (24:1) was added and then vortexed for 30 s. The sample mix was transferred to 2 ml phasemaker tubes (Invitrogen) and centrifuged at 4°C for 8 min at 16500 × *g*. A total of 300 μl of the upper phase was transferred to a new tube and RNA was precipitated by adding 500 μl of isopropanol and mixed by inversion. This was then centrifuged at 4°C for 4 min at 16500 × *g*. The supernatant was removed, and the RNA pellet was washed with 75% ice cold ethanol. The sample was centrifuged at 4°C for 4 min at 16500 × *g*, ethanol was removed, and the pellet was allowed to air dry before resuspending in 26 μl of nuclease free water. Concentration of RNA was determined using NanoDrop One^*c*^ (Thermo Fisher Scientific). To eliminate DNA contamination, RNA was treated with DNase1 (Invitrogen) and column purified using Zymo RNA Clean and Concentrate -5 (Zymo Research) following the manufacturer’s instructions. RNA integrity was determined by 1% agarose gel electrophoresis, and the RNA concentration and purity were determined using NanoDrop One^*c*^. First-strand cDNA was synthesized from 2 μg of total RNA using OligodT primers and Superscript III^TM^ reverse transcriptase (Invitrogen) according to the manufacturer’s instructions. qRT-PCR was performed using Power SYBR Green PCR Master Mix reagents (Applied Biosystems) (1X Power SYBER Green master mix, 250 nM of each primer, 6.25 ng of cDNA template in a total volume of 15 μl), in an optical 96-well plate. The Power SYBR Green qRT-PCR cycle conditions were carried using the Quant Studio 6 Real-Time PCR platform (Applied Biosystems) following the manufacturer’s instructions. Analysis of relative expression was carried out by the change in Ct, where the standard curve method was used to for relative transcript quantification normalized to *N. benthamina* glyceraldehyde 3-phosphate dehydrogenase (GAPDH) (Liu et al., 2012). Primers used for transcript analysis are listed in Supplementary Table S2.

### Protoplast Isolation and Transfection

Protoplast isolation from soybean cell suspension cultures was performed as previously described by Sultana et al. (2019). Protoplasts were quantified using a hemocytometer to calculate concentration along with fluorescein diacetate stain to determine cell viability. After protoplast isolation, protoplasts were kept on ice for 30 min prior to transfection. Protoplast transfection was performed in triplicate for each construct (*n* = 3), using 6 × 10^5^ protoplasts per ml following the robotic protocol from Sultana et al. (2019). Briefly, once protoplasts settled during incubation on ice, the supernatant was removed and replaced with MMg (0.4 M D-mannitol, 15 mM MgCl_2_, and 4 mM MES; pH 5.7). Plasmid DNA (10 μg) was pipetted into a well followed by 100 μl of protoplasts in MMg in a deep 96-well plate. An equal volume of 40% PEG solution (4 g of PEG 4000, 3 mL H_2_O, 2.5 ml of 0.8 M D-mannitol, and 1 ml of 1 M CaCl_2_) was added to each well. Then, the plate was transferred to a plate shaker and mixed at 1500 rpm for 10 s. The transformation mixture was then incubated at room temperature for 20 min with no agitation. After incubation, 500 μl of W5 solution (154 mM NaCl, 125 mM CaCl_2_, 5 mM KCl, and 2 mM MES) was added to each well and the plate was shaken at 1500 rpm for 10 s to terminate the reaction. Cells were allowed to settle for 30 min and 320 μl of supernatant was aspirated and discarded. The protoplast mixture was washed twice with 500 μl of WI (0.5 M D-Mannitol, 4 mM KCl, 4 mM MES; pH 5.7) and cells were allowed to settle for 30 min in between each wash in order to remove 500 μl of supernatant without disrupting the cells. Finally, the total 400 μl protoplast solution was transferred from 1 deep-well to 2 wells of a 96-well microplate (200 μl in each well) and incubated in the dark at room temperature for 24 h prior to microscopy.

### Confocal Microscopy and Standoff Detection

Soybean protoplasts and *N. benthamiana* leaf sections were observed using an Olympus Fluoview1200 confocal microscope (Olympus, Center Valley, PA, United States) to qualitatively determine the level of fluorescent protein gene expression. The fluorescent protein reporters mTag-BFP2, mEmerald and mScarlet-I were imaged using excitation (Ex)/emission (Em) wavelengths of 399/454, 487/509, and 569/593 nm, respectively. Chlorophyll autofluorescence was excited at 543 nm and detected at 667 nm. For comparison, images were acquired using the same laser parameters. For standoff detection, a recently developed fluorescence induced laser projector (FILP) was used to acquire images of agroinfiltrated plants expressing the fluorescent reporters according to parameters described previously (Rigoulot et al., 2019). mEmerald fluorescent images were acquired using the 465 nm excitation laser and 525/50 nm emission filter with 150 ms exposure.

### Statistical Analysis of Results

Statistical analysis was performed using IBM SPSS software (IBM Corp., Version 25.0). For analysis of fold change data or fluorescence spectral data at 509 nm, a one-way ANOVA was performed, followed by *post hoc* analysis using Tukey HSD. Analysis of continuous spectral data was performed using a one-way repeated measures ANOVA, which measures significance taking into account all data points along the spectrum. For qRT-PCR gene expression data, comparisons between groups were determined using independent samples *t*-test (Student’s *t*-test). For all statistical analysis a confidence level of *p* = 0.05 was used.

## Results

### Validation of Q-System in *N. benthamiana* and Soybean

Previous work determined that the QF transcriptional activator prevented regeneration of transgenic *Drosophila* leading to the development of the mutated, non-toxic, QF transcriptional activators QF2 and QF2w (Potter et al., 2010; Riabinina et al., 2015). To validate the function of QF2 and QF2w in plants, transient assays were performed using soybean protoplasts and agroinfiltration of *N. benthamiana* leaves. Initial experiments were performed using three 35S:Q:5xQUAS:mEmerald constructs, where Q represents either QF, QF2, or QF2w, along with the 5xQUAS:mEmerald negative control. Qualitative analysis of transgenic soybean protoplasts demonstrated strong mEmerald expression in all constructs containing the Q activator, whereas no expression was observed in the 5xQUAS:mEmerald negative control (Supplementary Figure S1). Based on the qualitative observations in soybean protoplasts, quantitative fluorescence data was collected in *N. benthamiana* using the same constructs relative to a 35S:mEmerald control. The 35S:QF construct showed a ∼4-fold amplification of signal relative to the control, whereas the 35S:QF2 and 35S:QF2w showed a 1.9 fold amplification of signal. In order to determine if the promoter driving QF has a significant role in the level of amplification, another set of constructs was tested in *N. benthamiana*, with the nopaline synthase (Nos) promoter driving the Q activator (QF, QF2, and QFw). Using the Nos promoter, an ∼8-fold amplification of signal was observed compared to the control expressing Nos:mEmerald (Figure 2). Similarly, a ∼6-fold amplification of signal was observed when QF2 or QF2w were expressed (Figure 2). From these data, despite the 35S promoter showing a 1.6-fold increase in mEmerald expression relative to the Nos promoter (Figure 2), the relative enhancement of signal from Nos:mEmerald to Nos:QF was 2X greater (8-fold vs. 4-fold) than 35S:mEmerald to 35S:QF. In addition, for both sets of constructs, the QF activator led to increased expression of the reporter relative to both QF2 and QF2w, with no significant difference in expression observed between QF2 and QF2w (Figure 2). This effect was more dramatic when the 35S:Q constructs were used, with QF showing ∼2.3-fold enhancement over QF2 and QF2w compared to only a 1.3-fold enhancement when the Nos:Q constructs were used. When the 35S:Q and Nos:Q constructs were normalized to the expression of Nos:mEmerald, the highest reporter signal was observed for the Nos:QF construct (*p* < 0.05), with no significant difference between 35S:QF, Nos:QF2, and Nos:QF2w constructs (Figure 2). The 35S:QF2 and 35S:QF2w constructs showed significantly lower expression than all other Q activators tested (*p* < 0.05).

**FIGURE 3:**
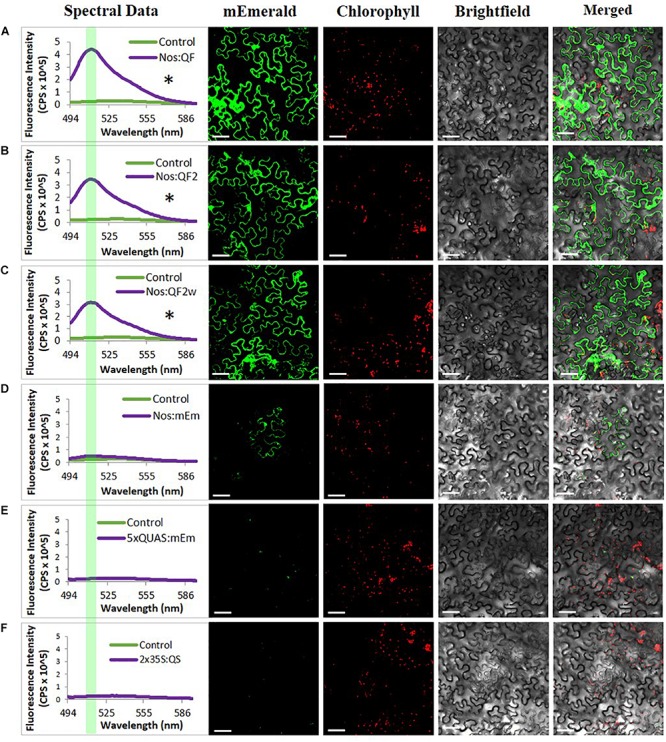
Signal amplification using Q-system variants QF, QF2, and QF2w. Spectral counts and confocal micrographs from **(A)** Nos:QF; **(B)** Nos:QF2; **(C)** Nos:QF2w; **(D)** Nos:mEmerald; **(E)** 5xQUAS:mEmerald; and **(F)** 2 × 35S:QS. Spectral data represent mEmerald emission of agroinfiltrated *N. benthamiana* leaves using the indicated constructs. With QF, QF2, and QF2w constructs, significant expression is observed relative to the empty vector control. The highlighted portion of the spectral data indicates peak mEmerald emission at 509 nm. Confocal micrographs allow for visual confirmation of the spectral data with the QF, QF2, and QF2w constructs easily observable. Statistical significance determined for all data points across spectrum using one-way repeated measures ANOVA, *post hoc* Tukey HSD). Asterisk (*) indicate significant difference of *p* < 0.05 compared to plants infiltrated with LBA4404 as a control and plants with the Nos:mEmerald construct. Data represent mean ± standard error of three independent experiments (*n* = 3). Three technical replicates were collected for each biological replicate to account for positional error.

In addition to the quantitative spectral data collected in Figure 2, confocal microscopy was conducted on agroinfiltrated *N. benthamiana* leaves and soybean protoplasts to visualize the expression of the reporters at the cell level using the Nos:Q:5xQUAS:mEmerald constructs (Figure 3 and Supplementary Figure S1). Observations of agroinfiltrated leaf sections showed strong fluorescence for all Nos:Q constructs relative to the Nos:mEmerald (Figure 3). This corresponded with the spectral data, where a significant increase (*p* < 0.05) in reporter expression was obtained for the Nos:Q constructs compared to the Nos:mEmerald control (Figure 3). These observations were consistent with soybean protoplast assays; where strong fluorescence was observed for the Nos:Q constructs when compared to Nos:mEmerald (Supplementary Figure S1). When compared to the 35S:Q constructs, there was no observable difference in fluorescence intensity from the Nos:Q constructs; however, both could be easily identified.

### Q-System as a Signal Amplifier for Standoff Detection and Controller for Metabolic Engineering in Plants

**FIGURE 4:**
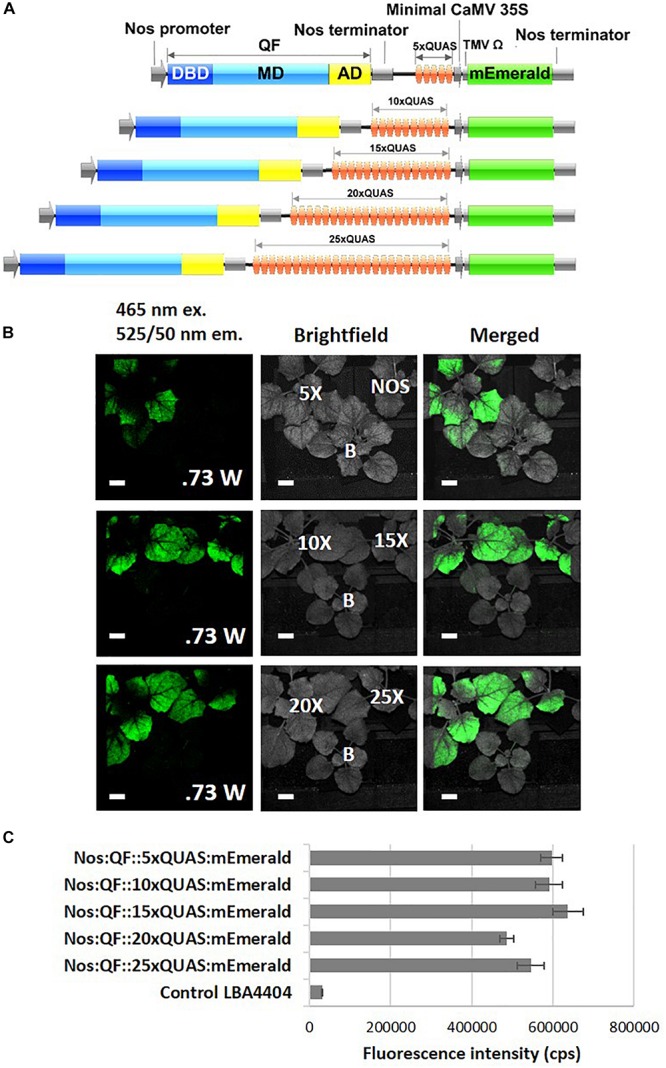
Effect of varying number of QUAS repeats on mEmerald emission. **(A)** Schematic of QUAS repeat constructs. **(B)** Images acquired using the FILP system show the ability to detect mEmerald from all QUAS repeats tested; however, the NOS:mEmerald construct without amplification by the Q-system could not be detected. The fluorescent images were acquired using a 150 ms exposure time. **(C)** Spectral analysis of mEmerald emission readings at 509 nm. Statistical significant differences between groups determined using one-way ANOVA with *post hoc* analysis using Tukey HSD: groups with different letters show a significant difference (*p* < 0.05). Error bars, standard error of mean. Scale bar: 2.5 cm. The brightfield image is labeled for each of the QUAS repeats tested, the empty vector negative control, labeled **(B)**, and the Nos:mEmerald positive control. Data represent mean ± standard error of three independent experiments (*n* = 3). Three technical replicates were collected for each biological replicate to account for positional error.

In order to evaluate the ability of the Q-system to significantly amplify the signal from a fluorescent reporter driven by a weak promoter, remote imaging (>3 m standoff) experiments were conducted. As indicated by spectral data, the Nos:QF:5xQUAS:mEmerald signal from agroinfiltrated leaves was clearly visible, while the Nos:mEmerald signal could not be detected by the fluorescence induced laser projector (FILP) system (Figure 4). To determine if further signal amplification could be achieved by increasing the number of QUAS repeats (5xQUAS, 10xQUAS, 15xQUAS, 20xQUAS, and 25xQUAS) leaf agroinfiltration was repeated with these constructs. Based on imaging with the FILP system, it was possible to visualize the fluorescent signal from all QUAS repeats at standoff, although there was no observable difference in signal between the constructs. Quantitative fluorescence spectroscopy data confirmed that there was no significant amplification in fluorescence between the QUAS repeats (*p* > 0.05) (Figure 4). These results were further confirmed at the cell level by confocal microscopy of agroinfiltrated leaf sections (Supplementary Figure S2).

**FIGURE 5:**
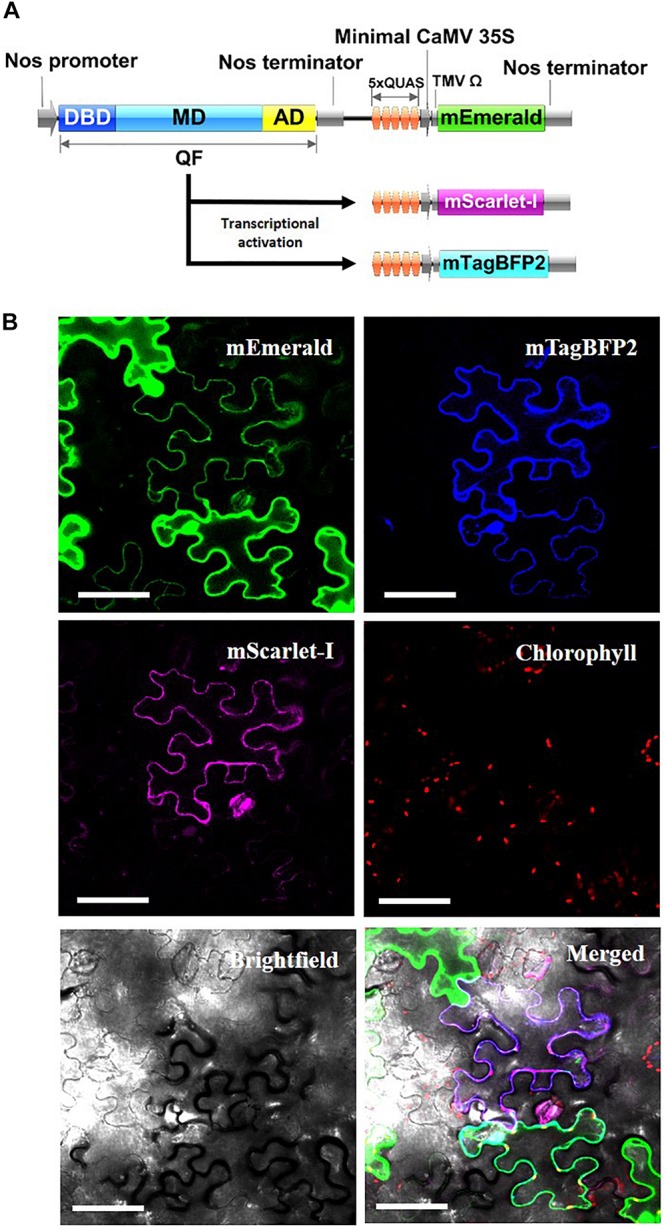
Simultaneous activation of multiple reporter genes using a single QF transcription factor. **(A)** Schematic of *N. benthamiana* transient expression assay where QF is only expressed on the Nos:QF:5xQUAS:mEmerald construct. All three constructs (5xQUAS:mTagBFP2, 5xQUAS:mScarlet-I, and Nos:QF:5xQUAS:mEmerald) were co-transformed to determine simultaneous activation of three distinct fluorescent proteins. Experiments were performed in triplicate (*n* = 3). **(B)** Confocal micrographs showing expression of three distinct fluorescent proteins controlled by the expression of QF on only a single construct. The intensity of mEmerald was decreased in cells expressing multiple fluorescent protein. Further, using this experimental design, cells could not express either mScarlet-I or mTagBFP2 unless the mEmerald construct was present. Chlorophyll autofluorescence was used as a control to set the initial laser threshold. Scale bar: 50 μm.

As another potential application, the Q-system was evaluated for the potential to simultaneously control the expression of multiple transgenes from a single QF activator. As such, Nos:QF:5xQUAS:mEmerald was co-infiltrated into *N. benthamiana* leaves with 5xQUAS:mTagBFP2 and 5xQUAS:mScarlet-I (Figure 5). In this experimental design, 3 distinct constructs were used for co-infiltration, with QF only expressed on a binary construct that also expressed mEmerald (Figure 5). As such, it was possible to observe cells singly expressing mEmerald; however, expression of mTagBFP2 or mScarlet-I required co-transformation with the binary Nos:QF:5xQUAS:mEmerald construct. Confocal microscopy images showed expression of all three fluorescent reporters within a single cell, confirming co-transfection (Figure 5). In addition to cells expressing all three fluorescent reporters, it was possible to observe multiple cells singly expressing mEmerald. It should be noted that cells expressing only mEmerald had increased fluorescence compared to cells that were expressing multiple reporters (Figure 5 and Supplementary Figure S3). Similarly, in no instances were mTagBFP2 and mScarlet-I observed in a cell without the presence of mEmerald.

### Suppression of the QF Transcription Factor by QS

**FIGURE 6:**
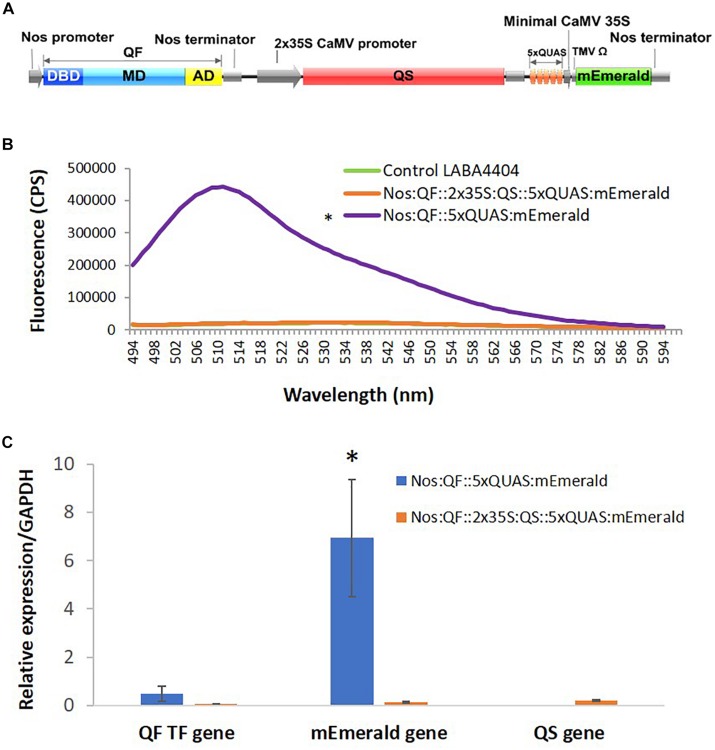
Suppression of QF by expression of QS. **(A)** Schematic of the Q-system suppression vector: Nos:QF:2 × 35S:QS:5xQUAS:mEmerald. DBD, DNA binding domain; MD, middle domain; AD, activation domain; QS, Q-system suppressor. **(B)** Spectral analysis of *N. benthamiana* transiently infected leaves expressing the mEmerald reporter. Statistical significance was determined for all data points across the spectrum using one-way repeated measures ANOVA, with *post hoc* analysis using Tukey HSD. Asterisk (*) indicate significant difference compared to plants infiltrated with LBA4404 as a control (*p* < 0.05). Data represent mean ± standard error of three independent experiments (*n* = 3). Three technical replicates were collected from each independent experiment to account for variability in the sample. **(C)** Variation in expression levels of mEmerald and QF in plants transiently infected with the Q-system activation construct (Nos:QF:5xQUAS:mEmerald) and Q-system QS suppression construct (Nos:QF:2 × 35S:QS:5xQUAS:mEmerald), as determined by qRT-PCR. The relative levels of the transcripts were normalized to expression of *N. benthamiana g*lyceraldehyde 3-phosphate dehydrogenase (GAPDH). There was no significant difference in the expression of the QF transcription factor between the constructs (*p* > 0.05); however, there was a significant difference (*p < 0.05) in mEmerald expression. This confirms that the reduced expression was due to suppression of QF by QS. qRT-PCR was performed on tissue collected from three independent experiments (*n* = 3), with three technical replicates collected per experiment.

Previous work on QF suppression by QS, in plant co-infiltration experiments, indicated strong but incomplete suppression (Reis et al., 2018). As such, a cassette was designed that contained all three components of the Q-system: the QF activator controlled by a Nos promoter, the QS suppressor controlled by a 2 × 35S promoter, and the 5xQUAS driving the expression of an mEmerald reporter gene (Figure 6). Compared to plants infiltrated with Nos:QF:5xQUAS:mEmerald, there was a 21.7-fold decrease in mEmerald expression in the Nos:QF:2 × 35S:QS:5xQUAS:mEmerald infiltrated plants. In fact, there was no significant difference between the construct containing QS and the control plants infiltrated with *Agrobacterium* alone (*p* > 0.05) (Figure 6). To confirm that the reduction in fluorescent signal was due to suppression by QS, qRT-PCR was performed to measure the expression of QF in the two constructs. Furthermore Figure 6 shows there was no significant difference in expression of QF between the two constructs (*p* > 0.05), although there was a significantly higher level of mEmerald expression (*p* < 0.05). Qualitative analysis by confocal microscopy and standoff detection confirmed this result, with no fluorescence observed at the cell level in agroinfiltration experiments, or with soybean protoplasts (Supplementary Figure S1).

## Discussion

When selecting an inducible transgene expression system, it is important to minimize off-target effects and maintain host plant growth and development. The main characteristics of a chemical-inducible systems include: (1) high specificity; (2) significant fold induction; (3) low basal expression in the absence of inducer; and (4) fast response time (Zuo and Chua, 2000). Based on studies in insects and mammalian cells, the Q-system has proven to be an excellent inducible system with high modularity (Potter et al., 2010; Riabinina et al., 2015, 2019). In plants, having a repressible binary expression system like the Q-system, would not only allow transgene expression systems to be highly flexible, but also allow the control and regulation of multiple genes with a single transactivator and repressible element.

The QF transcription factor was first reported to be functional in plants by Reis et al. (2018), where it was used with a conditional silencing suppression system designed from the potato leafroll virus. In this earlier work, it was demonstrated that the QF transcriptional activator was functional in plant transient assays, and that QS could suppress QF function, although not completely. In the present study, we expanded on this initial work and demonstrated the functionality of multiple QF variants in multiple plant species, the ability to control multiple genes with a single QF activator, and the potential of the Q-system to amplify the signal from a weak promoter for the purposes of remote detection. QF and its variants, QF2 and QF2w, were proven functional and effectively enhanced reporter gene expression under the control of a weak Nos and the 35S CaMV promoter (Figures 2, 3). *Agrobacterium* transient expression experiments with both the Nos and CaMV 35S driven Q-system transactivator variants indicated that the original QF gave the highest level of mEmerald emission under the Nos and 35S CaMV promoter. In contrast, weaker but comparable mEmerald expression levels were observed with the Nos:QF2 and Nos:QF2w variants (Figure 2). In *Drosophila*, a similar trend of lower but comparable level of reporter expression was observed for QF2w compared to QF2 (Riabinina et al., 2015). From these results, the QF activator and its variants appear to be more effective at amplification of reporter gene expression under the Nos promoter, than the 35S CaMV promoter. Since the original QF was shown to be lethal in *Drosophila* (Potter et al., 2010), it was expected that the original QF would also show similar levels of toxicity in plants. Based on the significantly lower levels of reporter gene expression obtained for the 35S Q-system variants (35S:QF2 and 35S:QF2w) compared to Nos Q-system variants (Nos:QF2 and Nos:QF2w), we hypothesize that saturation and potential toxicity on plant cells could be occurring in 35S:QF constructs. It was noted in studies on *Drosophila* that the QF variants may also have some level of toxicity (Riabinina and Potter, 2016). However, no toxicity was apparent in transient leaf infiltration or protoplast transfection studies. For further analysis of QF toxicity in plants, it would be ideal to study stably-transformed plants with a constitutive and inducible QF. Our current knowledge of QF toxicity suggests that the QF2 variant is currently the best Q activator option in plants. Since the QF2 and QF2w variants used in this study were codon optimized for studies in *Drosophila* (Potter et al., 2010; Riabinina and Potter, 2016), it is possible that some level of codon optimization for use of the Q-system in plants may lead to improved levels of reporter gene expression.

To further investigate the potential of the Q-system for signal amplification and tuning gene expression, the effect of increasing the number of QUAS repeats driving reporter gene expression was examined. Our results indicated that there was no significant difference in amplification of reporter genes as the number of QUAS repeats increased above 5xQUAS (Figure 4C). An increase in expression was expected in plants since previous studies in *Drosophila* showed that expression levels were fine-tuned and significantly increased as the number of QUAS repeats was increased from 5 to 10 (Pfeiffer et al., 2012; Shearin et al., 2014). However, these effector lines harboring varying numbers of QUAS repeats, also included tandem fusions of reporter genes (Shearin et al., 2014) controlled by additional regulatory elements that acted as translational enhancers (Pfeiffer et al., 2010). It should be noted that in the present work, the multiple 5xQUAS repeats were synthesized back-to-back without spacers inserted in between he 5xQUAS repeats. As such, a comprehensive analysis that varies the spacing between 5xQUAS repeats may yield greater information on the potential to further enhance the Q-system. Despite the inability to further amplify the reporter gene signal by varying the number of QUAS repeats, plants transiently infected with constructs containing varying QUAS repeats showed strong signal amplification in spectroscopic analysis (Figure 4C) and were visible with the FILP system (Figure 4C). These results indicate that QF-based amplification of weak promoters is sufficient to enhance reporter gene expression for remote detection. These observations validate the statistical analysis for spectral data obtained in Figure 4C; where there does not appear to be any significant difference in mEmerald fluorescence, detected by the FILP system, between constructs featuring greater than 5xQUAS repeats.

Another important aspect of an inducible expression system is its ability to coordinate the expression of multiple genes. Here, we show that with the Q-system, a single QF transcription factor was able to modulate expression of all three distinct fluorescent proteins (mEmerald, mTagBFP2, and mScarlet-I) within a single cell (Figure 5B). In cells where two or more fluorescent reporters were expressed, the intensity of mEmerald emission was lower (Figure 5B), likely due to competition of the QF transcription factor for binding to the 5xQUAS sequence driving the expression each fluorescent reporter. Based on these observations, in order to utilize the Q-system for regulating the expression of multiple genes, it is important to have optimal levels of QF expression based on the number of genes being regulated. This was apparent for the mScarlet-I gene where fluorescence was lower and difficult to detect in transient expression assays on both spectrofluorescence-based analysis and confocal imaging.

One advantage of the Q-system over other repressible binary systems, like the GAL4 system, is that expression of the QF transactivator can be suppressed by QS and then temporarily regulated by the presence of the small non-toxic molecule quinic acid (QA) (Potter et al., 2010; Riabinina et al., 2015). QA inhibits QS activity allowing QF to be reactivated to allow binding to the QUAS driving expression of a gene of interest (Riabinina and Potter, 2016). Although the mechanism of QS suppression of QF is relatively unknown, it is an essential component in the use of the Q-system as a repressible binary system for regulating transgene expression. As expected, in transient expression experiments, in both *N. benthamiana* and soybean protoplasts, QS was effective at tightly suppressing QF activity (Supplementary Figure S1). In addition, qRT-PCR data analysis showed a significant decrease in mEmerald gene expression for plants infiltrated with the QS suppression construct (Figure 6C).

The additional use of the inducer QA was shown to successfully derepress QF activity in *Drosophila* (Potter et al., 2010). However, in plants subsequent reactivation of the Q-system using QA is yet to be successfully reported. This repressive feature of the Q-system provides a unique tool for creating an inducible system in plants which can be reactivated by the use of QA or by removal of the QS suppressor. Furthermore, creating a split system, where the QF transcription activator is added only when activation is required provides another level for controlling gene expression. A recent study on the split-QF system, where split transactivator constructs, one harboring the DBD and the other the AD, were shown to be fully functional, repressible and inducible in *Drosophila* (Riabinina et al., 2019). It has also been shown that the Q-system components can be highly flexible and combined with other expression systems, such as GAL4 and Lex (Riabinina et al., 2015, 2019; Riabinina and Potter, 2016).

Collectively, we show that the Q-system components can function effectively in plants. Our results demonstrated activation of three reporter genes with a single QF transcription factor further supporting the Q-system as a promising platform in plant metabolic engineering. It can be effectively used in tuning the level of transgene expression in plants by simply changing the QF transcriptional activator variant to either the QF2 or QF2w depending on the level of gene amplification desired. Furthermore, it has the potential to provide a multidimensional tool kit which can be used for controlling and regulating transgene expression in myriad of ways, including its use for both tissue specific spatial regulation and temporal regulation of transgenes. Overall, the Q-system is highly flexible, adding new gene regulatory components for use in plant synthetic biology.

## Data Availability Statement

All datasets generated for this study are included in the article/Supplementary Material.

## Author Contributions

RP and SL wrote the manuscript. RP, LD, and AO designed the experiments. RP, SR, and MS performed the experiments for standoff detection. RP, LD, DR, M-AN, JL, and MP performed the experiments and collected the data for protoplasts and transient assays. RP and LD analyzed the data. CS and SL conceived of the study and its design and coordination, and assisted with interpretation of results and revisions to the manuscript. All authors read, contributed to improving text, and approved the final manuscript.

## Conflict of Interest

The authors declare that the research was conducted in the absence of any commercial or financial relationships that could be construed as a potential conflict of interest.

## Supplementary Material

FIGURE S1 Q-system variants expressing mEmerald in soybean protoplasts. Confocal micrographs from obtained 24–48 h post transformation. The Q-system construct used for transfection is indicated to the right of panel. Transfection was performed in triplicate (*n* = 3). Scale bar: 100 μm (10×), 50 μm (40×).

FIGURE S2 Effect of QUAS effector repeats on amplification of mEmerald. Spectral counts and confocal micrographs from **(A)** Nos:QF::5xQUAS:mEmerald; **(B)** Nos:QF::10xQUAS:mEmerald; **(C)** Nos:QF::15xQUAS:mEmerald; **(D)** Nos:QF::20xQUAS:mEmerald; **(E)** Nos:QF::25xQUAS:mEmerald; and **(F)** control plant infiltrated with LBA4404. Spectral analysis of transiently infected *N. benthamiana* leaves expressing the mEmerald reporter. Spectrofluorescence readings obtained 72 h post *Agrobacterium* infection. Statistical significance determined for all data points across spectrum using one-way repeated measures ANOVA, *post hoc* Tukey HSD). Asterisk (^∗^) indicates significant difference (*p* < 0.05) in expression when compared to plants infiltrated with LBA4404 as a control. Data represent mean ± standard error of three independent experiments (*n* = 3), with three technical replicates collected per experiment.

FIGURE S3 Confocal micrographs of co-infiltrated leaves where QF was present only on the Nos:QF:5xQUAS:mEmerald construct. The 5xQUAS:mTagBFP2 and 5xQUAS:mScarlet-I constructs require the initial construct in order to activate their respective fluorescent protein. Images showing simultaneous activation of the three distinct fluorescent proteins: mEmerald, mTagBFP2 and mScarlet-I when leaves were co-infiltrated with all three constructs. Three independent experiments were performed (*n* = 3), with three biological replicates per experiment. Scale bar: 50 μm (40×).

TABLE S1 Sequences of fragments used for vector construction.

TABLE S2 Primers used for qRT-PCR.
