# Supplementary figures and images for: The Q-System as a Synthetic Transcriptional Regulator in Plants
Source: Front Plant Sci. 2020 Mar 11;11:245. doi: 10.3389/fpls.2020.00245 (PMC7078239; doi:10.3389/fpls.2020.00245)

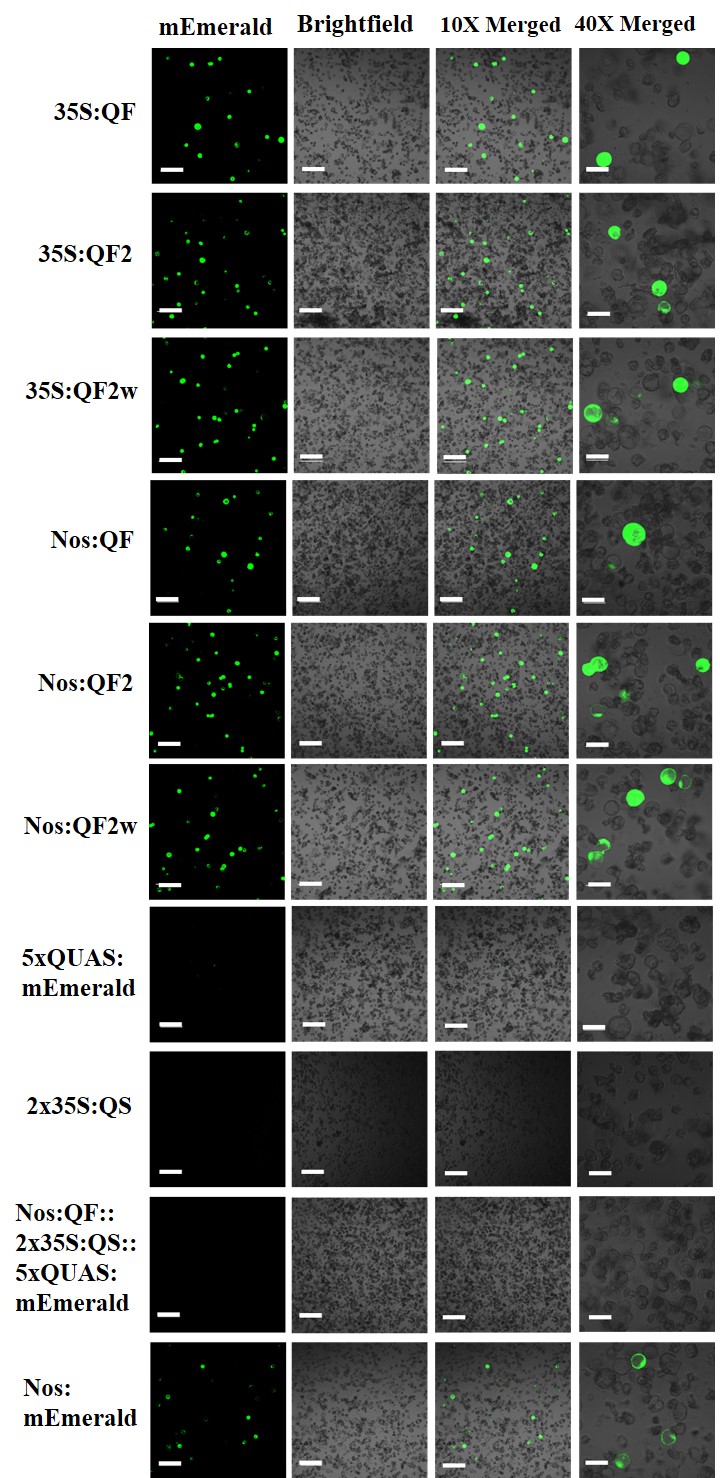

Supplement: FIGURE S1 — Q-system variants expressing mEmerald in soybean protoplasts. Confocal micrographs from obtained 24–48 h post transformation. The Q-system construct used for transfection is indicated to the right of panel. Transfection was performed in triplicate (n = 3). Scale bar: 100 μm (10×), 50 μm (40×). [file Image_1.JPEG]

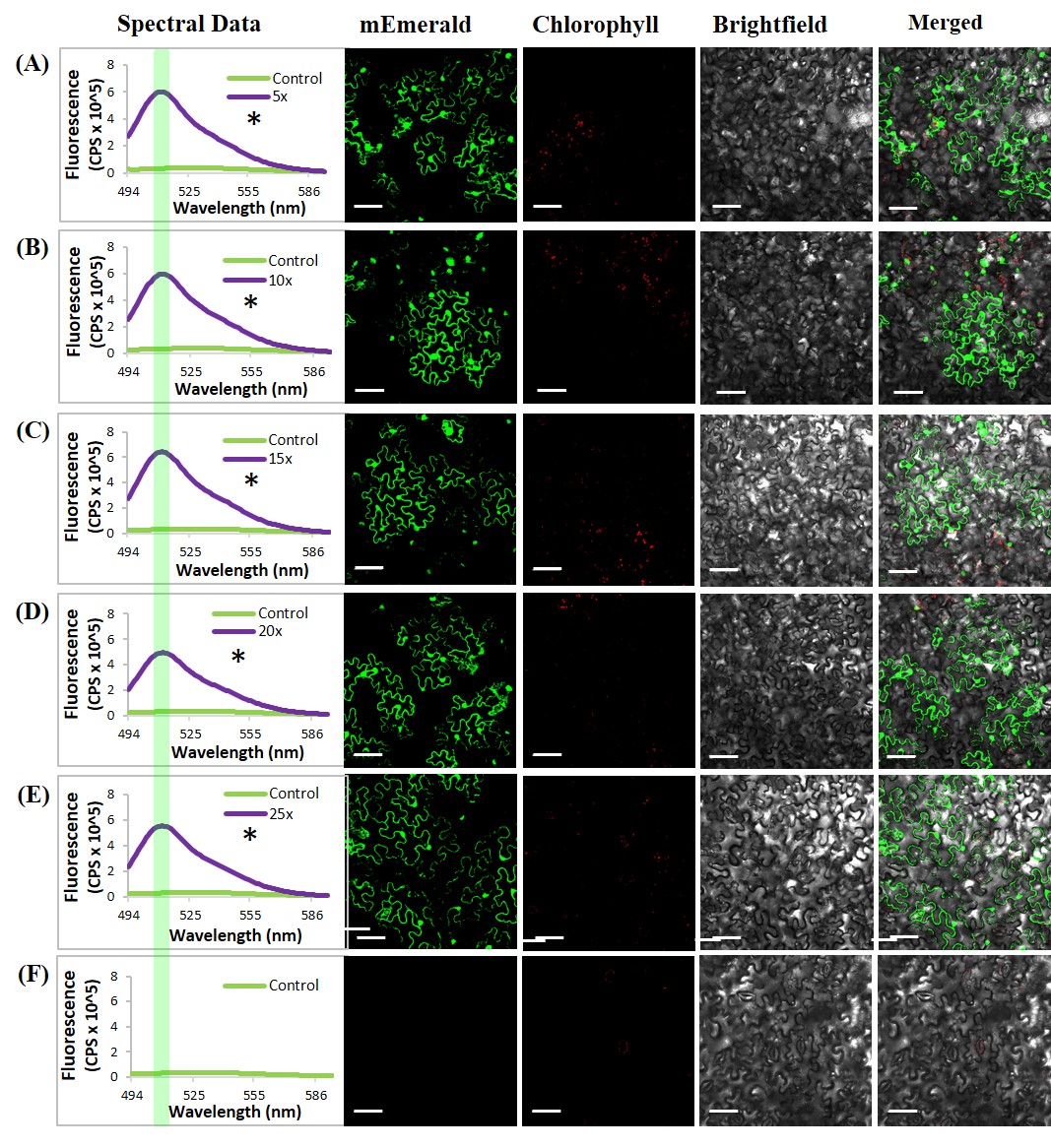

Supplement: FIGURE S2 — Effect of QUAS effector repeats on amplification of mEmerald. Spectral counts and confocal micrographs from (A) Nos:QF::5xQUAS:mEmerald; (B) Nos:QF::10xQUAS:mEmerald; (C) Nos:QF::15xQUAS:mEmerald; (D) Nos:QF::20xQUAS:mEmerald; (E) Nos:QF::25xQUAS:mEmerald; and (F) control plant infiltrated with LBA4404. Spectral analysis of transiently infected N. benthamiana leaves expressing the mEmerald reporter. Spectrofluorescence readings obtained 72 h post Agrobacterium infection. Statistical significance determined for all data points across spectrum using one-way repeated measures ANOVA, post hoc Tukey HSD). Asterisk (∗) indicates significant difference (p < 0.05) in expression when compared to plants infiltrated with LBA4404 as a control. Data represent mean ± standard error of three independent experiments (n = 3), with three technical replicates collected per experiment. [file Image_2.JPEG]

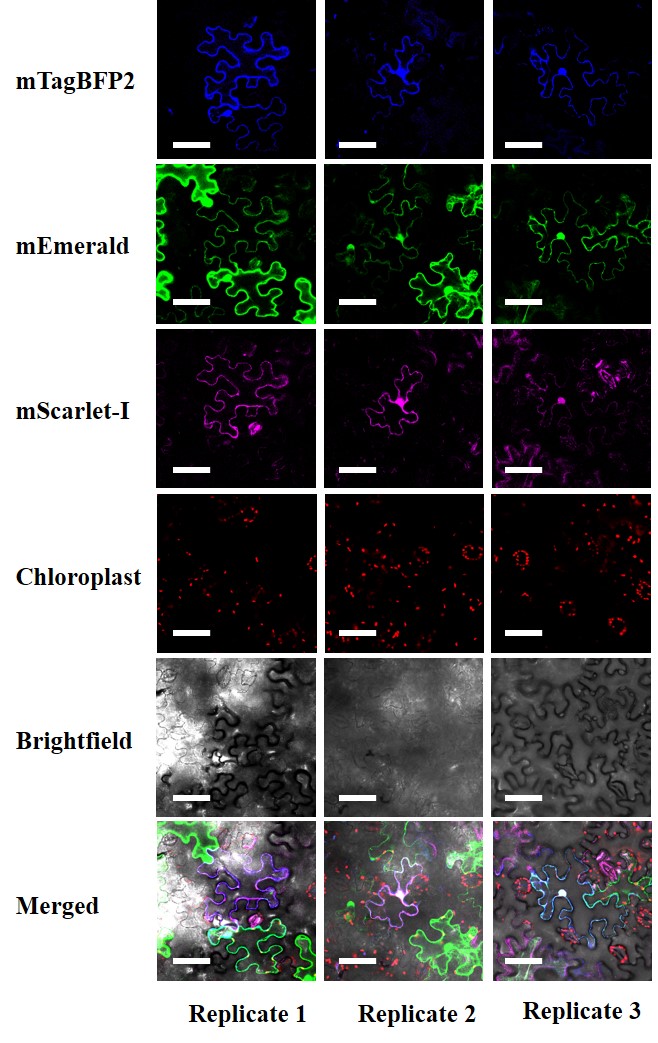

Supplement: FIGURE S3 — Confocal micrographs of co-infiltrated leaves where QF was present only on the Nos:QF:5xQUAS:mEmerald construct. The 5xQUAS:mTagBFP2 and 5xQUAS:mScarlet-I constructs require the initial construct in order to activate their respective fluorescent protein. Images showing simultaneous activation of the three distinct fluorescent proteins: mEmerald, mTagBFP2 and mScarlet-I when leaves were co-infiltrated with all three constructs. Three independent experiments were performed (n = 3), with three biological replicates per experiment. Scale bar: 50 μm (40×). [file Image_3.JPEG]
