# Supplementary material for: The Q-System as a Synthetic Transcriptional Regulator in Plants
Source: Front Plant Sci. 2020 Mar 11;11:245. doi: 10.3389/fpls.2020.00245 (PMC7078239; doi:10.3389/fpls.2020.00245)
Supplement: TABLE S1 — Sequences of fragments used for vector construction. [file Data_Sheet_1.docx]

| **Supplemental Table S1. Sequences of fragments used for vector construction** | |
| --- | --- |
| *Name of DNA Fragment* | *Sequence* |
| PacI-5xQUAS:mEmerald-AscI | ttaattaagaattcGGGTAATCGCTTATCCtcGGATAAACAATTATCCtcacGGGTAATCGCTTATCCgctcGGGTAATCGCTTATCCtcGGGTAATCGCTTATCCttgaagacctcctgcgcaagacccttcctctatataaggaagttcatttcatttggagaggactatttttacaacaattaccaacaacaacaaacaacaaacaacattacaattactatttacaattacaccatggtgagcaagggcgaggagctgttcaccggggtggtgcccatcctggtcgagctggacggcgacgtaaacggccacaagttcagcgtgtccggcgagggcgagggcgatgccacctacggcaagctgaccctgaagttcatctgcaccaccggcaagctgcccgtgccctggcccaccctcgtgaccaccttgacctacggcgtgcagtgcttcgcccgctaccccgaccacatgaagcagcacgacttcttcaagtccgccatgcccgaaggctacgtccaggagcgcaccatcttcttcaaggacgacggcaactacaagacccgcgccgaggtgaagttcgagggcgacaccctggtgaaccgcatcgagctgaagggcatcgacttcaaggaggacggcaacatcctggggcacaagctggagtacaactacaacagccacaaggtctatatcaccgccgacaagcagaagaacggcatcaaggtgaacttcaagacccgccacaacatcgaggacggcagcgtgcagctcgccgaccactaccagcagaacacccccatcggcgacggccccgtgctgctgcccgacaaccactacctgagcacccagtccaagctgagcaaagaccccaacgagaagcgcgatcacatggtcctgctggagttcgtgaccgccgccgggatcactctcggcatggacgagctgtacaagaggtgagtggcgcgcc |
| MfeI-Nos:QF-SpeI | caattggggtttctggagtttaatgagctaagcacatacgtcagaaaccattattgcgcgttcaaaagtcgcctaaggtcactatcagctagcaaatatttcttgtcaaaatgctccactgacgttccataaattcccctcggtatccaattagagtctcatattcactctcaatccaaataatctgcaggtctccctaggATGCCGCCTAAACGCAAGACACTCAATGCCGCTGCCGAAGCCAATGCCCACGCTGATGGCCATGCTGATGGCAATGCTGATGGTCATGTCGCTAACACTGCAGCAAGCAGCAACAACGCCCGTTTTGCGGACTTGACCAACATTGACACACCCGGCCTCGGCCCTACCACGACGACGTTACTTGTCGAGCCCGCTCGTTCGAAACGCCAGAGAGTCTCGAGGGCCTGTGATCAGTGTCGAGCTGCACGTGAAAAGTGTGATGGAATCCAGCCGGCTTGCTTCCCCTGTGTGTCGCAGGGCCGGTCGTGTACCTACCAGGCCAGTCCCAAGAAGCGAGGAGTCCAGACGGGCTACATCCGCACTCTCGAACTGGCTCTGGCTTGGATGTTCGAGAACGTTGCCCGCAGCGAGGACGCCCTCCACAATCTTTTGGTCCGTGATGCTGGCCAGGGCAGCGCTCTCCTGGTCGGCAAAGACTCGCCTGCTGCAGAACGCCTGCATGCAAGATGGGCGACGAGTCGAGTCAACAAAAGCATCACCCGTCTTCTCTCAGGTCAGGCCGCACAAGATCCATCTGAAGACGGCCAATCCCCGTCCGAAGACATAAATGTCCAAGATGCCGGGGCAAAGACATCCGACTTCCCTCATGCGCCTCACTTGACTTTCTCGGCGCCTAAGTCTAGTACAGCTGAGACACGCACTCTACCAGGCCCGGTCCGACCACCTATTTCGGCAAACACCCTGGAAAACAACCTTCAGCCAGATGGTACCGGGATAGGGAAGCTACCACCCAATCATTGGCGCCTGCTGGATATCTACTTTTCCTACACGCATTCTTGGCTCCCTATCCTCGAGAAGAAAGACATGTACCAAGCATTGTACCAGTACTCTGAACAAGGCTCGTTGCTTCCCTCTGCGAATGTCGAGTCTGGCGTTCATGCCGAGCTCTGGAGCGCGCTCGCCCTGGCGTCCTTCCAGGCTGCTGCTACTGCTGCATCGAGTGCTACGGGTCCAGCTTCAGCTGCTCATGGCCATGACAATGCCATCAATCCTTCACCTGCAGACATATCTGACACAGCCCGAAAGCTCATACCTTTGGAAAGCGGGCCGTTCCAGGTTCAGCACTGCAGAGCGTTGCTGCTTCTTTGTCTCGTAAGCCTTGGGCGGGATGATTGGGAGTCTGCTTGGTTGCTGGTTGGCTTTGCGGTCCGCGTCCTACTTGTTGTTCGCACCCAGTTGCCTCCTGATGATGACCGGCCACGACCAAGAATGCGTGCGCTGCTCGTCGCGTGCTTCATCGTGGATACCATTGTGTCTATGAGACACAACGTGCCGGCCCATCTCAAGCCAGACGACATTGCGGATCTGCCGTTACCTGAAGACGGTCAAGATCAATGGGAGCCGTGGACACCATGTGAGGGCTTAGGCGGTGAACACACCATGCTGCAAATGTTGAGGAACCCGGCATACCCTTTAAGCACATTCAACCACCTATATGGCGTGACCAAGCTGGTTGCTTTGGAGCTTCTGCCAAGAATACGAACATCTTCACAGAACGCTCCCTTGGAGTTCAGGTCGCGGTTGCAGCAGGTAATCGGCCACAATTCTCCCTTCAGCGTCTTTGTCCTTTCCCAGGATACAGCATCGGCTTTTGTGCCTACTGCATACCTTACCCGTACCGTTTATTTATGGGCAGCTGCCTTTTCTGAGCCTCTCAACGAACACTACTCGCATCTTCTGATCGAAACTCTTGATCAGTATCAGAAGCGGTTTGGTACATATGCAATCCCACCTCTGATCCCTTCTCTTCTAGACTCCCTTCTTGCTTTAAAGAAACAATCACATTCTTCAGAGCGGCATCGAAGGCACTTGGAAGAGCTTTTCCCCGCCTACTCCTCCATTTGGCCTCGGGGAGGCCGACACAGCAATACTGGCCTCCAACCCATACGACAACTTGAGCTTCCGCCGACTGCGACTGCCACTGCAAGTATCATGCCCCATGTCATGGAACAGCCCCTGTCAACGTCAATAAATCCGGTCAATGATCGGTTTAATGGAATACCGAATCCTACCCCCTACAATAGCGATGCGGCCCTAGACGCAATTACTCAAACCAATGACTACGGATCAGTCAATACCCATGGCATTCTCAGTACATATCCACCACCTGCCACCCACTTGAACGAGGCCTCGGTGGCCCTTGCGCCTGGAGGTGCTCCTCCCAGACCACCCCCGCCATACGTTGATAGTACAACGAACCATCCTCCTTACCATAGTAACCTCGTCCCCATGGCCAATTTCGGATATTCGACTGTAGACTATGATGCGATGGTGGATGATCTGGCATCGATCGAGTACACGGATGCGGTTGACGTTGATCCGCAGTTCATGACGAACCTCGGGTTTGTCCCAGGGTGTAATTTTAGCGATATCAACACGTATGAGCAATAGtaaactagt |
| MfeI-Nos:QF2-SpeI | caattggggtttctggagtttaatgagctaagcacatacgtcagaaaccattattgcgcgttcaaaagtcgcctaaggtcactatcagctagcaaatatttcttgtcaaaatgctccactgacgttccataaattcccctcggtatccaattagagtctcatattcactctcaatccaaataatctgcaggtctccctaggATGCCACCCAAGCGCAAAACGCTTAACGCTGCGGCTGAGGCTAACGCTCATGCCGACGGACACGCCGACGGAAACGCCGACGGACACGTGGCCAATACGGCCGCGTCCTCGAATAATGCGAGGTTCGCTGATCTCACTAACATCGATACTCCGGGTCTGGGACCCACAACTACGACCCTGCTCGTGGAACCAGCACGCTCAAAGCGTCAACGAGTGTCCCGCGCATGCGACCAGTGCCGTGCAGCCCGAGAGAAATGCGACGGAATACAGCCTGCGTGTTTCCCGTGCGTTTCCCAGGGAAGGTCCTGCACTTATCAGGCTTCGCCGAAAAAGAGGGGAGTTCAAACCGGTTATATTCGTACGCTGGAGCTCGCCCTCGCCTGGATGTTTGAAAATGTCGCGCGTTCCGAAGATGCCTTGCATAACCTCCTCGTCCGTGACGCCGGACAAGGATCAGCTCTGCTCGTTGGTAAAGATTCGCCGGCTGCCGAGCGACTCCATGCCCGTTGGGCTACTAGCCGTGTCAATAAGAGCATTACCCGCCTCCTCCGTCAGTTGGAGCTCCCTCCTACCGCCACGGCTACGGCCTCGATAATGCCGCACGTGATGGAGCAGCCTCTCAGTACCAGCATTAACCCCGTCAACGACCGCTTCAACGGTATTCCCAACCCCACTCCGTATAACTCCGATGCAGCTCTCGATGCTATCACTCAGACCAACGATTATGGAAGCGTAAATACACATGGTATCCTCTCTACTTACCCGCCACCGGCTACGCACCTTAATGAAGCTTCCGTCGCTCTCGCTCCCGGTGGCGCCCCCCCCCGACCGCCTCCTCCGTATGTTGACAGCACGACCAATCACCCGCCGTACCACTCGAATCTGGTTCCAATGGCGAACTTTGGTTACTCGACCGTTGATTACGATGCCATGGTTGACGATTTGGCTAGCATTGAATACACGGACGCTGTGGATGTCGACCCACAGTTTATGACCAATCTGGGATTCGTTCCTGGATGTAACTTCTCCGACATTAATACATACGAACAGTGATGAtaaactagt |
| MfeI-Nos:QF2w-SpeI | caattggggtttctggagtttaatgagctaagcacatacgtcagaaaccattattgcgcgttcaaaagtcgcctaaggtcactatcagctagcaaatatttcttgtcaaaatgctccactgacgttccataaattcccctcggtatccaattagagtctcatattcactctcaatccaaataatctgcaggtctccctaggATGCCACCCAAGCGCAAAACGCTTAACGCTGCGGCTGAGGCTAACGCTCATGCCGACGGACACGCCGACGGAAACGCCGACGGACACGTGGCCAATACGGCCGCGTCCTCGAATAATGCGAGGTTCGCTGATCTCACTAACATCGATACTCCGGGTCTGGGACCCACAACTACGACCCTGCTCGTGGAACCAGCACGCTCAAAGCGTCAACGAGTGTCCCGCGCATGCGACCAGTGCCGTGCAGCCCGAGAGAAATGCGACGGAATACAGCCTGCGTGTTTCCCGTGCGTTTCCCAGGGAAGGTCCTGCACTTATCAGGCTTCGCCGAAAAAGAGGGGAGTTCAAACCGGTTATATTCGTACGCTGGAGCTCGCCCTCGCCTGGATGTTTGAAAATGTCGCGCGTTCCGAAGATGCCTTGCATAACCTCCTCGTCCGTGACGCCGGACAAGGATCAGCTCTGCTCGTTGGTAAAGATTCGCCGGCTGCCGAGCGACTCCATGCCCGTTGGGCTACTAGCCGTGTCAATAAGAGCATTACCCGCCTCCTCCGTCAGTTGGAGCTCCCTCCTACCGCCACGGCTACGGCCTCGATAATGCCGCACGTGATGGAGCAGCCTCTCAGTACCAGCATTAACCCCGTCAACGACCGCTTCAACGGTATTCCCAACCCCACTCCGTATAACTCCGATGCAGCTCTCGATGCTATCACTCAGACCAACGATTATGGAAGCGTAAATACACATGGTATCCTCTCTACTTACCCGCCACCGGCTACGCACCTTAATGAAGCTTCCGTCGCTCTCGCTCCCGGTGGCGCCCCCCCCCGACCGCCTCCTCCGTATGTTGACAGCACGACCAATCACCCGCCGTACCACTCGAATCTGGTTCCAATGGCGAACTTTGGTTACTCGACCGTTGATTACGATGCCATGGTTGACGATTTGGCTAGCATTGAATACACGGACGCTGTGGATGTCGACCCACAGTTTATGACCAATCTGGGATTCGTTCCTGGATGTAACTTCTCCGACATTAATACATACAAAAAGAAGAAATGATGAtaaactagt |
| MfeI-35S:QF-SpeI | caattgtgagacttttcaacaaagggtaatatccggaaacctcctcggattccattgcccagctatctgtcactttattgtgaagatagtggaaaaggaaggtggctcctacaaatgccatcattgcgataaaggaaaggccatcgttgaagatgcctctgccgacagtggtcccaaagatggacccccacccacgaggagcatcgtggaaaaagaagacgttccaaccacgtcttcaaagcaagtggattgatgtgatatctccactgacgtaagggatgacgcacaatcccactatccttcgcaagacccttcctctatataaggaagttcatttcatttggagaggacggtctccctaggATGCCGCCTAAACGCAAGACACTCAATGCCGCTGCCGAAGCCAATGCCCACGCTGATGGCCATGCTGATGGCAATGCTGATGGTCATGTCGCTAACACTGCAGCAAGCAGCAACAACGCCCGTTTTGCGGACTTGACCAACATTGACACACCCGGCCTCGGCCCTACCACGACGACGTTACTTGTCGAGCCCGCTCGTTCGAAACGCCAGAGAGTCTCGAGGGCCTGTGATCAGTGTCGAGCTGCACGTGAAAAGTGTGATGGAATCCAGCCGGCTTGCTTCCCCTGTGTGTCGCAGGGCCGGTCGTGTACCTACCAGGCCAGTCCCAAGAAGCGAGGAGTCCAGACGGGCTACATCCGCACTCTCGAACTGGCTCTGGCTTGGATGTTCGAGAACGTTGCCCGCAGCGAGGACGCCCTCCACAATCTTTTGGTCCGTGATGCTGGCCAGGGCAGCGCTCTCCTGGTCGGCAAAGACTCGCCTGCTGCAGAACGCCTGCATGCAAGATGGGCGACGAGTCGAGTCAACAAAAGCATCACCCGTCTTCTCTCAGGTCAGGCCGCACAAGATCCATCTGAAGACGGCCAATCCCCGTCCGAAGACATAAATGTCCAAGATGCCGGGGCAAAGACATCCGACTTCCCTCATGCGCCTCACTTGACTTTCTCGGCGCCTAAGTCTAGTACAGCTGAGACACGCACTCTACCAGGCCCGGTCCGACCACCTATTTCGGCAAACACCCTGGAAAACAACCTTCAGCCAGATGGTACCGGGATAGGGAAGCTACCACCCAATCATTGGCGCCTGCTGGATATCTACTTTTCCTACACGCATTCTTGGCTCCCTATCCTCGAGAAGAAAGACATGTACCAAGCATTGTACCAGTACTCTGAACAAGGCTCGTTGCTTCCCTCTGCGAATGTCGAGTCTGGCGTTCATGCCGAGCTCTGGAGCGCGCTCGCCCTGGCGTCCTTCCAGGCTGCTGCTACTGCTGCATCGAGTGCTACGGGTCCAGCTTCAGCTGCTCATGGCCATGACAATGCCATCAATCCTTCACCTGCAGACATATCTGACACAGCCCGAAAGCTCATACCTTTGGAAAGCGGGCCGTTCCAGGTTCAGCACTGCAGAGCGTTGCTGCTTCTTTGTCTCGTAAGCCTTGGGCGGGATGATTGGGAGTCTGCTTGGTTGCTGGTTGGCTTTGCGGTCCGCGTCCTACTTGTTGTTCGCACCCAGTTGCCTCCTGATGATGACCGGCCACGACCAAGAATGCGTGCGCTGCTCGTCGCGTGCTTCATCGTGGATACCATTGTGTCTATGAGACACAACGTGCCGGCCCATCTCAAGCCAGACGACATTGCGGATCTGCCGTTACCTGAAGACGGTCAAGATCAATGGGAGCCGTGGACACCATGTGAGGGCTTAGGCGGTGAACACACCATGCTGCAAATGTTGAGGAACCCGGCATACCCTTTAAGCACATTCAACCACCTATATGGCGTGACCAAGCTGGTTGCTTTGGAGCTTCTGCCAAGAATACGAACATCTTCACAGAACGCTCCCTTGGAGTTCAGGTCGCGGTTGCAGCAGGTAATCGGCCACAATTCTCCCTTCAGCGTCTTTGTCCTTTCCCAGGATACAGCATCGGCTTTTGTGCCTACTGCATACCTTACCCGTACCGTTTATTTATGGGCAGCTGCCTTTTCTGAGCCTCTCAACGAACACTACTCGCATCTTCTGATCGAAACTCTTGATCAGTATCAGAAGCGGTTTGGTACATATGCAATCCCACCTCTGATCCCTTCTCTTCTAGACTCCCTTCTTGCTTTAAAGAAACAATCACATTCTTCAGAGCGGCATCGAAGGCACTTGGAAGAGCTTTTCCCCGCCTACTCCTCCATTTGGCCTCGGGGAGGCCGACACAGCAATACTGGCCTCCAACCCATACGACAACTTGAGCTTCCGCCGACTGCGACTGCCACTGCAAGTATCATGCCCCATGTCATGGAACAGCCCCTGTCAACGTCAATAAATCCGGTCAATGATCGGTTTAATGGAATACCGAATCCTACCCCCTACAATAGCGATGCGGCCCTAGACGCAATTACTCAAACCAATGACTACGGATCAGTCAATACCCATGGCATTCTCAGTACATATCCACCACCTGCCACCCACTTGAACGAGGCCTCGGTGGCCCTTGCGCCTGGAGGTGCTCCTCCCAGACCACCCCCGCCATACGTTGATAGTACAACGAACCATCCTCCTTACCATAGTAACCTCGTCCCCATGGCCAATTTCGGATATTCGACTGTAGACTATGATGCGATGGTGGATGATCTGGCATCGATCGAGTACACGGATGCGGTTGACGTTGATCCGCAGTTCATGACGAACCTCGGGTTTGTCCCAGGGTGTAATTTTAGCGATATCAACACGTATGAGCAATAGtaaactagt |
| MfeI-35S:QF2-SpeI | caattgtgagacttttcaacaaagggtaatatccggaaacctcctcggattccattgcccagctatctgtcactttattgtgaagatagtggaaaaggaaggtggctcctacaaatgccatcattgcgataaaggaaaggccatcgttgaagatgcctctgccgacagtggtcccaaagatggacccccacccacgaggagcatcgtggaaaaagaagacgttccaaccacgtcttcaaagcaagtggattgatgtgatatctccactgacgtaagggatgacgcacaatcccactatccttcgcaagacccttcctctatataaggaagttcatttcatttggagaggacggtctccctaggATGCCACCCAAGCGCAAAACGCTTAACGCTGCGGCTGAGGCTAACGCTCATGCCGACGGACACGCCGACGGAAACGCCGACGGACACGTGGCCAATACGGCCGCGTCCTCGAATAATGCGAGGTTCGCTGATCTCACTAACATCGATACTCCGGGTCTGGGACCCACAACTACGACCCTGCTCGTGGAACCAGCACGCTCAAAGCGTCAACGAGTGTCCCGCGCATGCGACCAGTGCCGTGCAGCCCGAGAGAAATGCGACGGAATACAGCCTGCGTGTTTCCCGTGCGTTTCCCAGGGAAGGTCCTGCACTTATCAGGCTTCGCCGAAAAAGAGGGGAGTTCAAACCGGTTATATTCGTACGCTGGAGCTCGCCCTCGCCTGGATGTTTGAAAATGTCGCGCGTTCCGAAGATGCCTTGCATAACCTCCTCGTCCGTGACGCCGGACAAGGATCAGCTCTGCTCGTTGGTAAAGATTCGCCGGCTGCCGAGCGACTCCATGCCCGTTGGGCTACTAGCCGTGTCAATAAGAGCATTACCCGCCTCCTCCGTCAGTTGGAGCTCCCTCCTACCGCCACGGCTACGGCCTCGATAATGCCGCACGTGATGGAGCAGCCTCTCAGTACCAGCATTAACCCCGTCAACGACCGCTTCAACGGTATTCCCAACCCCACTCCGTATAACTCCGATGCAGCTCTCGATGCTATCACTCAGACCAACGATTATGGAAGCGTAAATACACATGGTATCCTCTCTACTTACCCGCCACCGGCTACGCACCTTAATGAAGCTTCCGTCGCTCTCGCTCCCGGTGGCGCCCCCCCCCGACCGCCTCCTCCGTATGTTGACAGCACGACCAATCACCCGCCGTACCACTCGAATCTGGTTCCAATGGCGAACTTTGGTTACTCGACCGTTGATTACGATGCCATGGTTGACGATTTGGCTAGCATTGAATACACGGACGCTGTGGATGTCGACCCACAGTTTATGACCAATCTGGGATTCGTTCCTGGATGTAACTTCTCCGACATTAATACATACGAACAGTGATGAtaaactagt |
| MfeI-35S:QF2w-SpeI | caattgtgagacttttcaacaaagggtaatatccggaaacctcctcggattccattgcccagctatctgtcactttattgtgaagatagtggaaaaggaaggtggctcctacaaatgccatcattgcgataaaggaaaggccatcgttgaagatgcctctgccgacagtggtcccaaagatggacccccacccacgaggagcatcgtggaaaaagaagacgttccaaccacgtcttcaaagcaagtggattgatgtgatatctccactgacgtaagggatgacgcacaatcccactatccttcgcaagacccttcctctatataaggaagttcatttcatttggagaggacggtctccctaggATGCCACCCAAGCGCAAAACGCTTAACGCTGCGGCTGAGGCTAACGCTCATGCCGACGGACACGCCGACGGAAACGCCGACGGACACGTGGCCAATACGGCCGCGTCCTCGAATAATGCGAGGTTCGCTGATCTCACTAACATCGATACTCCGGGTCTGGGACCCACAACTACGACCCTGCTCGTGGAACCAGCACGCTCAAAGCGTCAACGAGTGTCCCGCGCATGCGACCAGTGCCGTGCAGCCCGAGAGAAATGCGACGGAATACAGCCTGCGTGTTTCCCGTGCGTTTCCCAGGGAAGGTCCTGCACTTATCAGGCTTCGCCGAAAAAGAGGGGAGTTCAAACCGGTTATATTCGTACGCTGGAGCTCGCCCTCGCCTGGATGTTTGAAAATGTCGCGCGTTCCGAAGATGCCTTGCATAACCTCCTCGTCCGTGACGCCGGACAAGGATCAGCTCTGCTCGTTGGTAAAGATTCGCCGGCTGCCGAGCGACTCCATGCCCGTTGGGCTACTAGCCGTGTCAATAAGAGCATTACCCGCCTCCTCCGTCAGTTGGAGCTCCCTCCTACCGCCACGGCTACGGCCTCGATAATGCCGCACGTGATGGAGCAGCCTCTCAGTACCAGCATTAACCCCGTCAACGACCGCTTCAACGGTATTCCCAACCCCACTCCGTATAACTCCGATGCAGCTCTCGATGCTATCACTCAGACCAACGATTATGGAAGCGTAAATACACATGGTATCCTCTCTACTTACCCGCCACCGGCTACGCACCTTAATGAAGCTTCCGTCGCTCTCGCTCCCGGTGGCGCCCCCCCCCGACCGCCTCCTCCGTATGTTGACAGCACGACCAATCACCCGCCGTACCACTCGAATCTGGTTCCAATGGCGAACTTTGGTTACTCGACCGTTGATTACGATGCCATGGTTGACGATTTGGCTAGCATTGAATACACGGACGCTGTGGATGTCGACCCACAGTTTATGACCAATCTGGGATTCGTTCCTGGATGTAACTTCTCCGACATTAATACATACAAAAAGAAGAAATGATGAtaaactagt |
| EcoRI-QS-AscI | GAATTCATGAACACCATCCCGGCACGCCATGTCGGGGATGTCGCCGCCCGCGATCCTCTACCTCTACCGCACATATCATCCTCCGTCGCCAGCGGCATGAAGCGTTCCTTCGCAACCATGGCCATGCTCTACAACGACACTGGCAACAGCAACGATGTCGGTGCCCATGCCAGGCGACCACCACGAACCCTCTCCAATAGTCGGAGCACTTCCGCCCACAGAGTACCTCTAGGCTCTTGGTCGGCGCCCAATTCCCCGCCCCGCCGTGCGCTGCCGCATCATCCCATCACCGCCAGCTTCGATCCCGATGCTTCCATTGTTATCGCCGGCATTCGTGGCGCTGGCAAGTCTACGCTGGCCATCATGGCATCTACCGCCATGAAGCGCAAGATAGTCGACCTGGAATCCGAGTTCCATCATCTTACTGGCTTGTCTAGTTCCAGCTACAAGAAGACACACGGCCCGGTCGACTATGGGAGGCGCCAGATCGCCATCTTGCAGAACATCTTGAATCTGCACAGGACCCGCGCCATTCTCGTCTGCTCTTGGCTGGAGCGGGATGTGCAGGCCATGTTGCAGGATTTCAGCGTGTCTAATCCTGTCATTTACGTTCTGCGCGATGCCAAAGCTATCGAGGCCCATCTGAAGGGATACGACAAGTCCAAAGTTGGCACCCTCCTTGATGCCACCAGTACTGTCCTTCGCCGCTGCACCCGTTTCGAGTTCTTCAACGTCTCCGAAGAGAACCTGGACACCCACTCCGCTTCAACATCACCACCTGCTGTTCCGGACCAGCGGCATACCGCGCCGTATCTAACGCTTAAACGAGCCGAGCGCCACTTCCTCAAATTTCTCTCCTTGATTCTACCCAAGGGGACCATACCTTTTGTCGAGTCCGCCTTTCCCCTGGCTTCCGTCCCCGTTGAACAGCGCCGCTTTACCTACGCCCTCGCCTTGCCTGTATCTGCCTTGCTCGACAAAGGCGTCGATATCCAAGAGCTTGATGTCGGTGTAGACGCAATCGAGATCATTGTAGACGATCTTGCAACGAGCGAATCCGGCCCAACGAGCCCCTTGGGTCTTGCGCCCCACCGAGCGAGCGAGATCAGTCGTGTTGTAGGCGAAATCAGGAGGGACACAGTGATCCCCATCATTCTGCACGTGGTCTTTCCAGAAAGAGCGCTATATGAAGAAGCTCTGCTCGCGCTTTACATGACTTACCTGAACCATGCCTTAAGGCTTGCGCCAGATTATCTTACGGTCGATCTGGGGCTCGATTCTGGCTTGCTTGGGCAACTAACCACCGTTCAAGGAACCACCAAGGTCATCGGCAATAAACAACTTGCAGAGGTCAATTCGCCGCGCTGGGGGGATCCATCTTGGTTACAAGCCTATGAAAAGGCCCAGAATACAGGATGCGACTTGGTAAGGTTGACCAGACCGGCTTCAAATCCCCGGGACAACACAGACATTCGGCAGTTCCACGTTGCTGTAGAGGCCGTCGGGGGTCCAAGGCTCCCATTTATTGCTTACAACACAGGACGCCTAGGTCGGACATCGATGTGTTTTAACGAGATCCTGACTCCAGTTACACCAGTGCCTACCAAGGAGGATGCAATCGGGCTCCGCAATCCAGCCCATCGCTATCTCCAGCCTCCGCTCACGGCTCTGGAAGCAACACAGGCTCTCTACTCGGCATTTGTCCACGACCCAATGAAGCTGTATGTCTTTGGCGCAAATGTGGGATATAGCTTGTCCCCAGCCATGCACAATGCCGCACTCAAGGCCTGTGGCATTCCACACCATTACAAGCCCCTTTCCACAGCAAACATCGGGACTTTGCGCGAGGTTATCAGCGATCCGCAGTTTGCTGGAGCCTCGGTCGGCCTGCCGTTTAAGGTGGAAATCATCAGCCTCACACACTCGCTGAGCCGGCACGCGAAAGCCATCGGAGCCGTCAACACCTTGATTCCGGTACGACACCTTACCGCGGACGGTGGAATACCGGACGAGGTGTCCATGTTCAACAATATCAGCCAAGCCGGCGCTGTCAGAGCTCTCTACGGCGAGAACACGGATTGGATTGGTATCCGAGCCTGCCTTCGCCGCGGTTTATCGCCCGCCAATGCCGTGAGATCAACAAGCACTGGTCTTGTCATCGGCGCTGGCGGAATGGCTAGGGCAGCTGTCTATGCCATGCTTCAACTGGGAGTCAAGAAGATTTTGATCTTTAACCGAACATTTGCTAATGCCGAGAAGCTGGTTCTACACTTCGAGAACCTGTTGGTCAGAGACGCATTGCCTCTGTTGAGCACAGGGCCAAGATCCCACGACAACACCTGTTTTCACATCATTCGATCTCGAGACGATCCGCTCCCAGAAAACTTCAAAAACCCGACCATGATCGTTTCCTGCATACCGACACACACAGTGGACAACACCCCTGACCCTGAATTTACTGTGCCTTTGCACTGGCTCGACAACCCCACTGGCGGCATTGTACTAGAACTCGACTACAAATGTCTCACATCACCCTTGCTCGAACAAACACGACGCGAGGCTCACAGAGGCTGGGTCGCAATGGATGGACTTGACCTCTTGCCAGAACAAGGGTTTGCCCAATTTGAACTGTTCACCGGGCGGCGAGCACCTCGTCGCTTGATGAGGCGCGAGGTTTTGCGAGCATACCCAGATGATCAAGCAAAATCTCATACCGCGCAGTTACAGCCTCGCCTCAACGGAATTGCAACGCAAATATCTTGATAAGGCGCGCC |
| SpeI-2X35S-QS-PacI | ACTAGTCCGATCGTTCAAACATTTGGCAATAAAGTTTCTTAAGATTGAATCCTGTTGCCGGTCTTGCGATGATTATCATATAATTTCTGTTGAATTACGTTAAGCATGTAATAATTAACATGTAATGCATGACGTTATTTATGAGATGGGTTTTTATGATTAGAGTCCCGCAATTATACATTTAATACGCGATAGAAAACAAAATATAGCGCGCAAACTAGGATAAATTATCGCGCGCGGTGTCATCTATGTTACTAGATCGGGAATCCTCGAGGAGATTAGCCTTTTCAATTTCAGAAAGAATGCTAACCCACAGATGGTTAGAGAGGCTTACGCAGCAGGTCTCATCAAGACGATCTACCCGAGCAATAATCTCCAGGAAATCAAATACCTTCCCAAGAAGGTTAAAGATGCAGTCAAAAGATTCAGGACTAACTGCATCAAGAACACAGAGAAAGATATATTTCTCAAGATCAGAAGTACTATTCCAGTATGGACGATTCAAGGCTTGCTTCACAAACCAAGGCAAGTAATAGAGATTGGAGTCTCTAAAAAGGTAGTTCCCACTGAATCAAAGGCCATGGAGTCAAAGATTCAAATAGAGGACCTAACAGAACTCGCCGTAAAGACTGGCGAACAGTTCATACAGAGTCTCTTACGACTCAATGACAAGAAGAAAATCTTCGTCAACATGGTGGAGCACGACACACTTGTCTACTCCAAAAATATCAAAGATACAGTCTCAGAAGACCAAAGGGCAATTGAGACTTTTCAACAAAGGGTAATATCCGGAAACCTCCTCGGATTCCATTGCCCAGCTATCTGTCACTTTATTGTGAAGATAGTGGAAAAGGAAGGTGGCTCCTACAAATGCCATCATTGCGATAAAGGAAAGGCCATCGTTGAAGATGCCTCTGCCGACAGTGGTCCCAAAGATGGACCCCCACCCACGAGGAGCATCGTGGAAAAAGAAGACGTTCCAACCACGTCTTCAAAGCAAGTGGATTGATGTGATATCTCCACTGACGTAAGGGATGACGCACAATCCCACTATCCTTCGCAAGACCCTTCCTCTATATAAGGAAGTTCATTTCATTTGGAGAGAACACGGGGGACTCTAGAGGATCCAAGGAGATATAACAATGAACACCATCCCGGCACGCCATGTCGGGGATGTCGCCGCCCGCGATCCTCTACCTCTACCGCACATATCATCCTCCGTCGCCAGCGGCATGAAGCGTTCCTTCGCAACCATGGCCATGCTCTACAACGACACTGGCAACAGCAACGATGTCGGTGCCCATGCCAGGCGACCACCACGAACCCTCTCCAATAGTCGGAGCACTTCCGCCCACAGAGTACCTCTAGGCTCTTGGTCGGCGCCCAATTCCCCGCCCCGCCGTGCGCTGCCGCATCATCCCATCACCGCCAGCTTCGATCCCGATGCTTCCATTGTTATCGCCGGCATTCGTGGCGCTGGCAAGTCTACGCTGGCCATCATGGCATCTACCGCCATGAAGCGCAAGATAGTCGACCTGGAATCCGAGTTCCATCATCTTACTGGCTTGTCTAGTTCCAGCTACAAGAAGACACACGGCCCGGTCGACTATGGGAGGCGCCAGATCGCCATCTTGCAGAACATCTTGAATCTGCACAGGACCCGCGCCATTCTCGTCTGCTCTTGGCTGGAGCGGGATGTGCAGGCCATGTTGCAGGATTTCAGCGTGTCTAATCCTGTCATTTACGTTCTGCGCGATGCCAAAGCTATCGAGGCCCATCTGAAGGGATACGACAAGTCCAAAGTTGGCACCCTCCTTGATGCCACCAGTACTGTCCTTCGCCGCTGCACCCGTTTCGAGTTCTTCAACGTCTCCGAAGAGAACCTGGACACCCACTCCGCTTCAACATCACCACCTGCTGTTCCGGACCAGCGGCATACCGCGCCGTATCTAACGCTTAAACGAGCCGAGCGCCACTTCCTCAAATTTCTCTCCTTGATTCTACCCAAGGGGACCATACCTTTTGTCGAGTCCGCCTTTCCCCTGGCTTCCGTCCCCGTTGAACAGCGCCGCTTTACCTACGCCCTCGCCTTGCCTGTATCTGCCTTGCTCGACAAAGGCGTCGATATCCAAGAGCTTGATGTCGGTGTAGACGCAATCGAGATCATTGTAGACGATCTTGCAACGAGCGAATCCGGCCCAACGAGCCCCTTGGGTCTTGCGCCCCACCGAGCGAGCGAGATCAGTCGTGTTGTAGGCGAAATCAGGAGGGACACAGTGATCCCCATCATTCTGCACGTGGTCTTTCCAGAAAGAGCGCTATATGAAGAAGCTCTGCTCGCGCTTTACATGACTTACCTGAACCATGCCTTAAGGCTTGCGCCAGATTATCTTACGGTCGATCTGGGGCTCGATTCTGGCTTGCTTGGGCAACTAACCACCGTTCAAGGAACCACCAAGGTCATCGGCAATAAACAACTTGCAGAGGTCAATTCGCCGCGCTGGGGGGATCCATCTTGGTTACAAGCCTATGAAAAGGCCCAGAATACAGGATGCGACTTGGTAAGGTTGACCAGACCGGCTTCAAATCCCCGGGACAACACAGACATTCGGCAGTTCCACGTTGCTGTAGAGGCCGTCGGGGGTCCAAGGCTCCCATTTATTGCTTACAACACAGGACGCCTAGGTCGGACATCGATGTGTTTTAACGAGATCCTGACTCCAGTTACACCAGTGCCTACCAAGGAGGATGCAATCGGGCTCCGCAATCCAGCCCATCGCTATCTCCAGCCTCCGCTCACGGCTCTGGAAGCAACACAGGCTCTCTACTCGGCATTTGTCCACGACCCAATGAAGCTGTATGTCTTTGGCGCAAATGTGGGATATAGCTTGTCCCCAGCCATGCACAATGCCGCACTCAAGGCCTGTGGCATTCCACACCATTACAAGCCCCTTTCCACAGCAAACATCGGGACTTTGCGCGAGGTTATCAGCGATCCGCAGTTTGCTGGAGCCTCGGTCGGCCTGCCGTTTAAGGTGGAAATCATCAGCCTCACACACTCGCTGAGCCGGCACGCGAAAGCCATCGGAGCCGTCAACACCTTGATTCCGGTACGACACCTTACCGCGGACGGTGGAATACCGGACGAGGTGTCCATGTTCAACAATATCAGCCAAGCCGGCGCTGTCAGAGCTCTCTACGGCGAGAACACGGATTGGATTGGTATCCGAGCCTGCCTTCGCCGCGGTTTATCGCCCGCCAATGCCGTGAGATCAACAAGCACTGGTCTTGTCATCGGCGCTGGCGGAATGGCTAGGGCAGCTGTCTATGCCATGCTTCAACTGGGAGTCAAGAAGATTTTGATCTTTAACCGAACATTTGCTAATGCCGAGAAGCTGGTTCTACACTTCGAGAACCTGTTGGTCAGAGACGCATTGCCTCTGTTGAGCACAGGGCCAAGATCCCACGACAACACCTGTTTTCACATCATTCGATCTCGAGACGATCCGCTCCCAGAAAACTTCAAAAACCCGACCATGATCGTTTCCTGCATACCGACACACACAGTGGACAACACCCCTGACCCTGAATTTACTGTGCCTTTGCACTGGCTCGACAACCCCACTGGCGGCATTGTACTAGAACTCGACTACAAATGTCTCACATCACCCTTGCTCGAACAAACACGACGCGAGGCTCACAGAGGCTGGGTCGCAATGGATGGACTTGACCTCTTGCCAGAACAAGGGTTTGCCCAATTTGAACTGTTCACCGGGCGGCGAGCACCTCGTCGCTTGATGAGGCGCGAGGTTTTGCGAGCATACCCAGATGATCAAGCAAAATCTCATACCGCGCAGTTACAGCCTCGCCTCAACGGAATTGCAACGCAAATATCTTGATAAGGCGCGCCCGATCGTTCAAACATTTGGCAATAAAGTTTCTTAAGATTGAATCCTGTTGCCGGTCTTGCGATGATTATCATATAATTTCTGTTGAATTACGTTAAGCATGTAATAATTAACATGTAATGCATGACGTTATTTATGAGATGGGTTTTTATGATTAGAGTCCCGCAATTATACATTTAATACGCGATAGAAAACAAAATATAGCGCGCAAACTAGGATAAATTATCGCGCGCGGTGTCATCTATGTTACTAGATCTTAATTAA |
| EcoRI-10xQUAS-BbsI | GAATTCGGGTAATCGCTTATCCTCGGATAAACAATTATCCTCACGGGTAATCGCTTATCCGCTCGGGTAATCGCTTATCCTCGGGTAATCGCTTATCCTTGGTAATCGCTTATCCTCGGATAAACAATTATCCTCACGGGTAATCGCTTATCCGCTCGGGTAATCGCTTATCCTCGGGTAATCGCTTATCCTTGAAGACCTCCTGC |
| EcoRI-15xQUAS-BbsI | GAATTCGGGTAATCGCTTATCCTCGGATAAACAATTATCCTCACGGGTAATCGCTTATCCGCTCGGGTAATCGCTTATCCTCGGGTAATCGCTTATCCTTGGTAATCGCTTATCCTCGGATAAACAATTATCCTCACGGGTAATCGCTTATCCGCTCGGGTAATCGCTTATCCTCGGGTAATCGCTTATCCTTGGTAATCGCTTATCCTCGGATAAACAATTATCCTCACGGGTAATCGCTTATCCGCTCGGGTAATCGCTTATCCTCGGGTAATCGCTTATCCTTGAAGACCTCCTGC |
| EcoRI-20xQUAS-BbsI | GAATTCGGGTAATCGCTTATCCTCGGATAAACAATTATCCTCACGGGTAATCGCTTATCCGCTCGGGTAATCGCTTATCCTCGGGTAATCGCTTATCCTTGGTAATCGCTTATCCTCGGATAAACAATTATCCTCACGGGTAATCGCTTATCCGCTCGGGTAATCGCTTATCCTCGGGTAATCGCTTATCCTTGGTAATCGCTTATCCTCGGATAAACAATTATCCTCACGGGTAATCGCTTATCCGCTCGGGTAATCGCTTATCCTCGGGTAATCGCTTATCCTTGGTAATCGCTTATCCTCGGATAAACAATTATCCTCACGGGTAATCGCTTATCCGCTCGGGTAATCGCTTATCCTCGGGTAATCGCTTATCCTTGAAGACCTCCTGC |
| EcoRI-25xQUAS-BbsI | GAATTCGGGTAATCGCTTATCCTCGGATAAACAATTATCCTCACGGGTAATCGCTTATCCGCTCGGGTAATCGCTTATCCTCGGGTAATCGCTTATCCTTGGTAATCGCTTATCCTCGGATAAACAATTATCCTCACGGGTAATCGCTTATCCGCTCGGGTAATCGCTTATCCTCGGGTAATCGCTTATCCTTGGTAATCGCTTATCCTCGGATAAACAATTATCCTCACGGGTAATCGCTTATCCGCTCGGGTAATCGCTTATCCTCGGGTAATCGCTTATCCTTGGTAATCGCTTATCCTCGGATAAACAATTATCCTCACGGGTAATCGCTTATCCGCTCGGGTAATCGCTTATCCTCGGGTAATCGCTTATCCTTGGTAATCGCTTATCCTCGGATAAACAATTATCCTCACGGGTAATCGCTTATCCGCTCGGGTAATCGCTTATCCTCGGGTAATCGCTTATCCTTGAAGACCTCCTGC |

| **Supplemental Table S2. Primers used for qRT-PCR** | | |
| --- | --- | --- |
| **Gene**​ | **Primer Sequence (5'-3')**​ | |
|  | **Forward**​ | **Reverse**​ |
| mEmerald ​ | AAGGGCATCGACTTCAAGGA                   ​ | ATGCCGTTCTTCTGCTTGTC  ​ |
| GAPDH​ | AGCTCAAGGGAATTCTCGATG​ | AACCTTAACCATGTCATCTCCC​ |
| QF ​ | CTATGATGCGATGGTGGATGA                 ​ | ATTACACCCTGGGACAAACC                  ​ |
| QS​ | GCGAGATCAGTCGTGTTGTAG  ​ | GCGAGCAGAGCTTCTTCATATAG​ |
